# Synthesis of 3-substituted isoxazolidin-4-ols using hydroboration–oxidation reactions of 4,5-unsubstituted 2,3-dihydroisoxazoles

**DOI:** 10.3762/bjoc.16.112

**Published:** 2020-06-16

**Authors:** Lívia Dikošová, Júlia Laceková, Ondrej Záborský, Róbert Fischer

**Affiliations:** 1 Institute of Organic Chemistry, Catalysis and Petrochemistry, Slovak University of Technology in Bratislava, Radlinského 9, 81237 Bratislava, Slovak Republic

**Keywords:** 2,3-dihydroisoxazoles, diastereoselectivity, heterocycles, hydroboration–oxidation, isoxazolidin-4-ols

## Abstract

Isoxazolidines represent a very important class of N/O-containing heterocycles used as the key intermediates in the synthesis of more complex cyclic and acyclic compounds, including various biologically active molecules. Here, we present a fast and highly stereoselective approach towards both C-3/4-*cis* and C-3/4-*trans* isomers of 3-substituted isoxazolidin-4-ols. The strategy relies on a highly regio- and *trans*-stereoselective hydroboration–oxidation reaction of the 4,5-unsubstituted 2,3-dihydroisoxazoles with basic hydrogen peroxide. The consecutive oxidation/reduction route, sequentially employing Dess–Martin periodinane and ʟ-selectride, is used for the inversion of the C-3/4-*trans* relative configuration of the isoxazolidine ring. The significance of the method lies in its variability and applicability to a concise synthesis of various 4-hydroxyisoxazolidines, starting from the readily available *C*-alkyl/aryl-nitrones. The resemblance to 3-hydroxypyrrolidines certainly makes the 4-hydroxyisoxazolidines important and valuable structural fragments in drug discovery.

## Introduction

2,3-Dihydroisoxazoles (often referred to as 4-isoxazolines) represent a very important class of N/O-containing heterocycles used as the key intermediates in the synthesis of more complex cyclic and acyclic compounds, including various biologically active molecules [1–6].

In this regard, we have recently developed new methods for the preparation of 4,5-unsubstituted 2,3-dihydroisoxazoles in moderate to very good yields, starting from readily available 5-acetoxy- and 5-hydroxyisoxazolidines [7–8]. Their reactivity in electrophilic addition reactions allows for a straightforward introduction of a hydroxy group at the C-4 position of the resulting isoxazolidines by means of dihydroxylation [9–10] and epoxidation [11–12] reactions. Regarding the stereochemistry, almost all of the realized additions proceed with an excellent *trans* stereoselectivity relative to the substituent at C-3, giving isoxazolidine-4,5-diols and isoxazolidinyl epoxides with a C-3/4-*trans* relative configuration.

**Figure 1:**
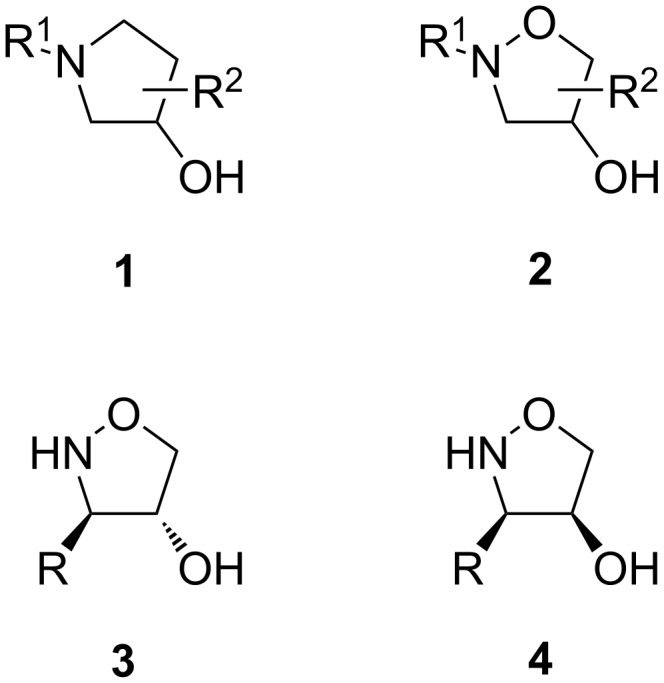
3-Substituted isoxazolidin-4-ols resembling 3-hydroxypyrrolidines.

The resemblance between 3-hydroxypyrrolidines **1** and 4-hydroxyisoxazolidines **2** (Figure 1) makes the latter valuable structural fragments in drug discovery [13–18]. Up to now, this class of compounds was commonly prepared using intramolecular nucleophilic substitution reactions of properly substituted hydroxylamines [19–20] and *N*-hydroxyphthalimides [21–24] as well as by Tamao oxidations of 4-silylisoxazolidines [25–26]. Very recently, Tomkinson et al. described a simple and effective method for an intramolecular oxyamination of allylic *N*-tosyl hydroxylamines using malonoyl peroxide, providing N-tosylated 3-aryl-substituted 4-hydroxyisoxazolidines in a highly stereoselective manner in favor of the C-3/4-*trans* isomers [27]. We believe that the 3-substituted isoxazolidin-4-ols, represented by the general structures of the *trans* and the *cis* stereoisomer **3** and **4** (Figure 1), would be of particular interest in modern medicinal chemistry as eventual heterocyclic substructures.

## Results and Discussion

**Scheme 1:**
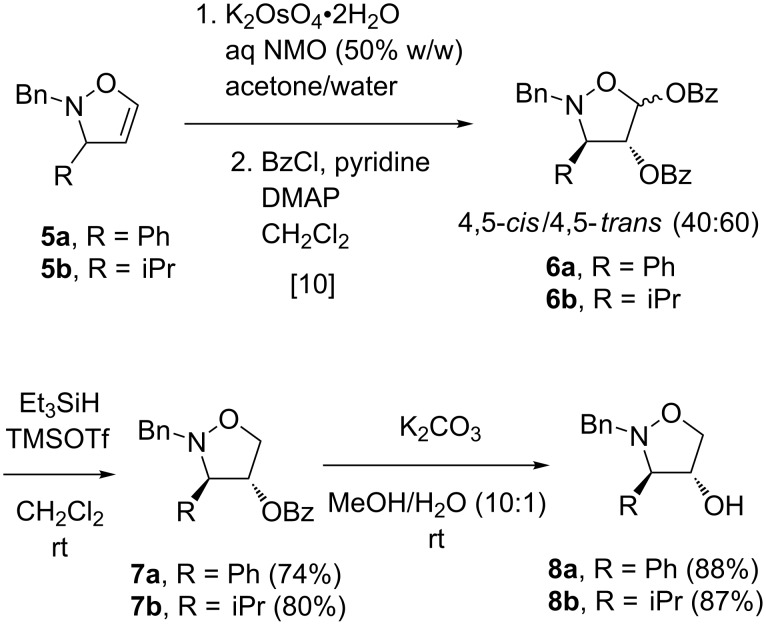
Synthetic approach towards isoxazolidin-4-ols via the regioselective reductive cleavage of the C5–O bond.

At the beginning of our investigations, we envisaged that the above-mentioned 3-substituted 4-hydroxyisoxazolidines bearing no substituent at C5 could be readily obtained through the reductive cleavage of benzoylated isoxazolidines, employing the Lewis acid-catalyzed S_N_ reaction with triethylsilane as the hydride source (Scheme 1). For this reason, the benzoates **6a** and **6b** were readily prepared from the corresponding 2,3-dihydroisoxazoles **5a** and **5b**, respectively, according to our procedure [7,10]. The compounds **5a** and **5b** were first converted into the isoxazolidine-4,5-diols by the treatment with potassium osmate/*N*-methylmorpholine *N*-oxide (NMO). The dihydroxylation reactions proceeded with an excellent *trans* selectivity with respect to the substituent at the C-3 carbon atom. The obtained products were benzoylated with benzoyl chloride/pyridine in the presence of DMAP to give the fully benzoylated isoxazolidine-4,5-diols **6a** and **6b**, which were subsequently treated with Et_3_SiH (3 equiv) and TMSOTf (2 equiv) in anhydrous CH_2_Cl_2_ at room temperature for 2 h [28]. To our delight, already the first attempts afforded the isoxazolidines **7a** and **7b** in very good 74% and 80% yield, respectively. Finally, their debenzoylation with K_2_CO_3_ in aqueous methanol gave the desired isoxazolidin-4-ols **8a** (88% yield) and **8b** (87% yield).

Although the obtained results clearly showed the applicability of the reductive cleavage of anomeric isoxazolidinyl carboxylates in the synthesis of the respective 5-unsubstituted 4-hydroxyisoxazolidines, this pioneering approach mainly suffered from a large number of synthetic steps starting from 2,3-dihydroisoxazoles, leading to the target alcohols. As a consequence, we assumed that the hydroboration–oxidation reaction of 2,3-dihydroisoxazoles would be an excellent way to prevent this obstacle. Recently, Kang et al. [29] reported the first hydroboration–oxidation reaction of the 5-substituted 4-isoxazolines even though the access to 4-hydroxyisoxazolidines by the treatment of boronic ester-substituted isoxazolidines with basic hydrogen peroxide has previously been described [30–31].

**Scheme 2:**
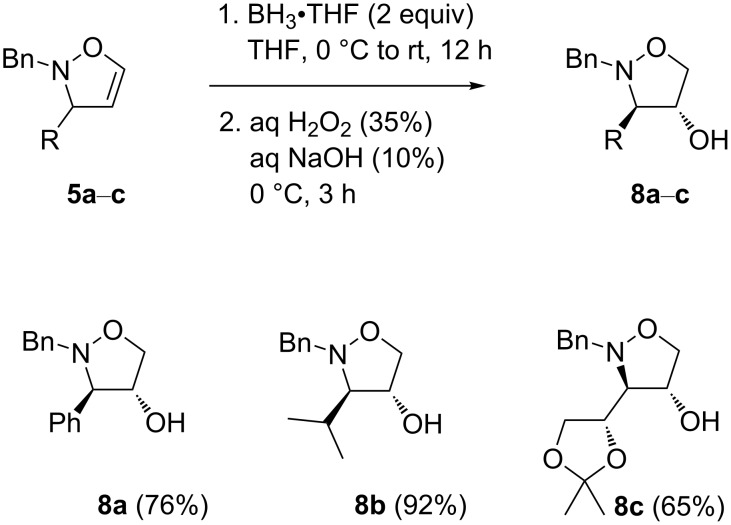
Hydroboration-oxidation of 4,5-unsubstituted 2,3-dihydroisoxazoles.

To start with, the phenyl-substituted 2,3-dihydroisoxazole **5a** was chosen as the starting substrate. After optimizing Kang's reaction conditions in terms of the borane amount, reaction temperature, and reaction time, we were able to prepare the 4-hydroxyisoxazolidine **8a** in the best yield of 76% (Scheme 2). Firstly, 2 equivalents of the BH_3_·THF complex were added at 0 °C, and the reaction was further conducted at room temperature for 12 h. Regarding the following oxidation step, after the disappearance of the starting material, 10% aqueous NaOH and 35% aqueous H_2_O_2_ were added at 0 °C dropwise as slowly as possible. It turned out that a patient addition of both NaOH (aq) and H_2_O_2_ (aq) accompanied by intense cooling is a crucial part of the procedure, preventing the formation of undesired byproducts, which can arise from an oxidation at the nitrogen atom, leading to the opening of the isoxazolidine ring. After stirring for 3 h at 0 °C, ethyl acetate was added to the reaction mixture, and stirring was continued at 0 °C for additional 10 min. Afterwards, the reaction was worked up and the product was isolated by flash column chromatography (FCC). Subsequently, the optimized reaction conditions were successfully applied for the 2,3-dihydroisoxazoles **5b** and **5c** (Scheme 2), the providing desired isoxazolidin-4-ols **8b** and **8c** in 92% and 65% yield, respectively.

All hydroborations proceeded with the exclusive formation of isoxazolidines with a hydroxy group in the C-4 position. We assume that the excellent regioselectivity is unambiguously caused by electronic effects since the endocyclic oxygen atom donates electrons to the C=C double bond, developing a negative partial charge at C-4. The existence of 4-hydroxy regioisomers was established by comparison of the ^1^H NMR spectrum of **8a** with the already reported values for the known 5-hydroxyisoxazolidine possessing the same substituents at the N2 and C-3 atom [32]. The main differences between both regioisomers can be observed in the chemical shifts of the H-4 and H-5 protons. The signals for the H-4 proton at 4.48 ppm and for the H-5 protons at 3.81 and 4.11 ppm clearly indicate the formation of the 4-hydroxy derivative, whereas in the 5-hydroxy regioisomer, the signals corresponding to the H-4 protons are shifted to lower values (2.08–3.30 ppm), and the signal for the H-5 proton can be found at 5.50 ppm due to the effect of the two oxygen atoms bound to the C5 carbon atom.

**Figure 2:**
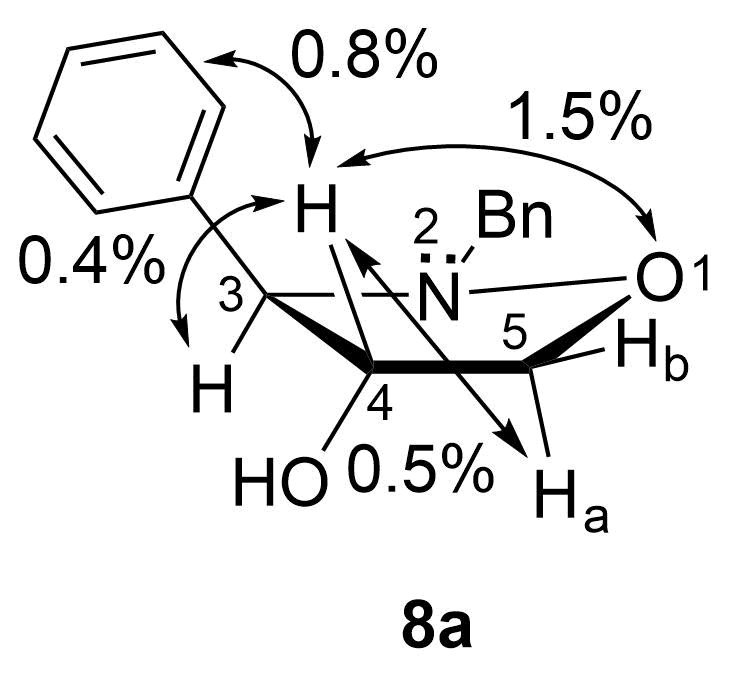
Selected NOE enhancements observed in the isoxazolidin-4-ol *trans*-**8a**. The arrows show the NOESY correlations.

Regarding the stereochemistry, the borane attack on the C=C double bond of the 2,3-dihydroisoxazole occurs from the sterically less hindered side, which is opposite relatively to the phenyl substituent at the C-3 carbon. The proposed C-3/4-*trans* relative configuration was confirmed by NOESY 1D experiments for **8a** (Figure 2), and the results obtained were later compared with those measured for the respective C-3/4-*cis* isomer. For **8a**, the irradiation of the H-4 proton resulted in a stronger enhancement of the signal corresponding to the pseudoequatorial proton H-5b (1.5%), whereas a weak NOE was observed for H-3 (0.4%) and the pseudoaxial H-5a proton (0.5%). Furthermore, the protons of the phenyl ring at C-3 were also affected (0.8%). The stereochemical results are consistent with our previous findings on the direct dihydroxylation and epoxidation reactions of 4,5-unsubstituted 2,3-dihydroisoxazoles [9–12].

**Scheme 3:**
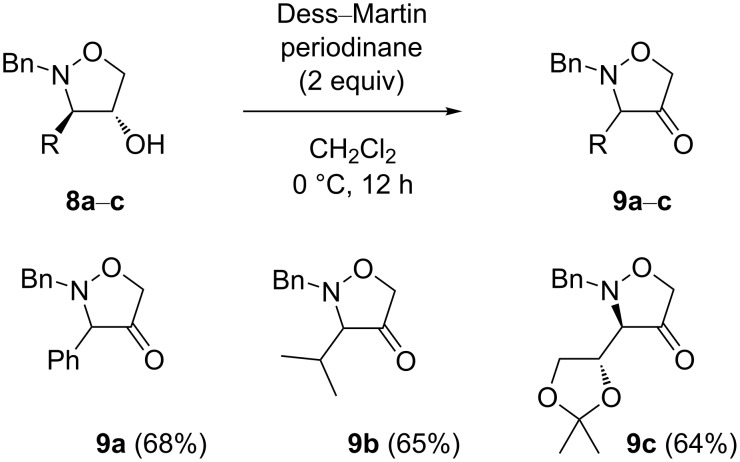
Dess-Martin oxidation of isoxazolidin-4-ols to ketones.

To invert the relative C-3/4-*trans* stereochemistry, the isoxazolidin-4-ols **5a**–**c** were first oxidized to the corresponding ketones (Scheme 3). Dess–Martin periodinane was chosen as the oxidizing agent [33–34] as our primary choice, pyridinium dichromate, prove to be insufficiently effective even at an elevated temperature. The reaction in anhydrous dichloromethane at 0 °C led to the desired isoxazolidin-4-ones **9a**–**c** in moderate isolated yields of 64–68%. The optimization of the reaction conditions showed that 2 equivalents of the oxidizing agent were necessary to bring the reaction to completion. Even though the ketones **9a**–**c** were isolated by FCC on silica gel in a pure form, they decomposed gradually if kept for a longer time, even at a lower temperature.

The structures of **9a**–**c** were definitely confirmed on the basis of ^13^C NMR spectra. The chemical shifts in the range of 210.8–215.0 ppm for the carbonyl carbon atom clearly indicated the presence of a ketone.

**Scheme 4:**
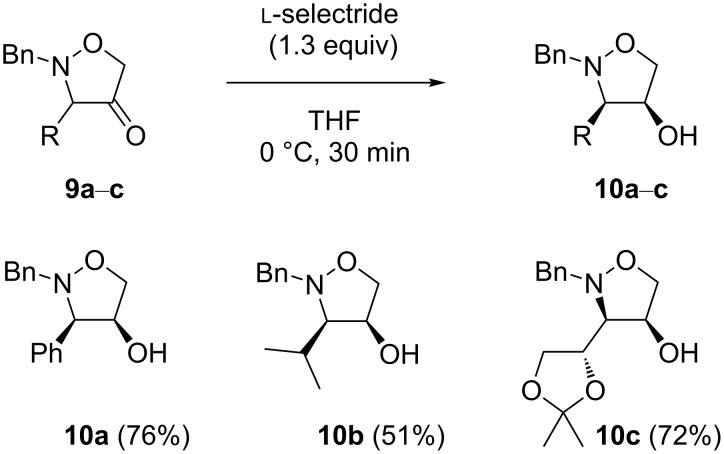
Inversion of the relative configuration of the isoxazolidine ring.

**Figure 3:**
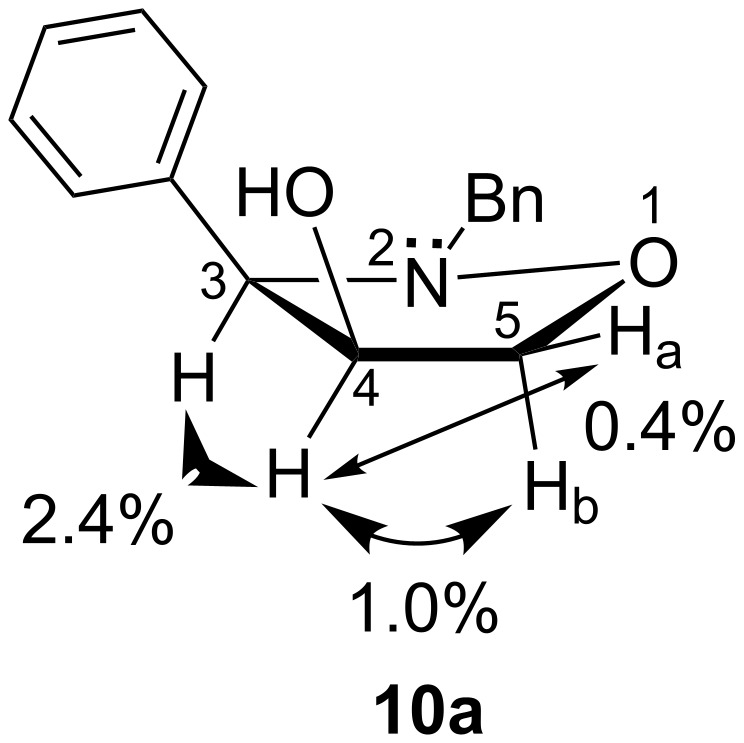
Selected NOE enhancements observed in the isoxazolidin-4-ol *cis*-**10a**. The arrows show the NOESY correlations.

With the isoxazolidin-4-ones **9a**–**c** in hand, we were ready to examine the intended stereoselective reduction of cyclic ketones with the aim to obtain the 4-hydroxyisoxazolidines with a relative C-3/4-*cis* configuration. Whereas the initial attempts of the reduction of **9a** with lithium borohydride resulted in a poor stereoselectivity (70:30 in favor of the desired *cis* isomer), the use of bulky, sterically hindered ʟ-selectride in anhydrous THF at 0 °C led to the formation of the *cis* product exclusively (Scheme 4) [35–36], and the C-3/4-*cis* 4-hydroxyisoxazolidine **10a** was isolated in 76% yield. Its relative configuration was confirmed by means of NOESY 1D experiments (Figure 3). Not surprisingly, the NOE between the protons H-3 and H-4 was significantly stronger (2.4%) than in the respective *trans* isomer **8a**.

The C-3/4-*cis* isoxazolidin-4-ols **10b** and **10c** were obtained from the ketones **9b** and **9c** in a similar manner in satisfying (51%) and good yield (72%), respectively (Scheme 4).

According to the literature, the N-debenzylation by a Pd-catalyzed hydrogenolysis in methanol with formic acid as the hydrogen source [37–38] should allow the access to N-deprotected isoxazolidines. Unfortunately, all attempts to remove the benzyl group directly from the model isoxazolidine **8b** resulted only in a reductive cleavage of the N–O bond.

**Scheme 5:**
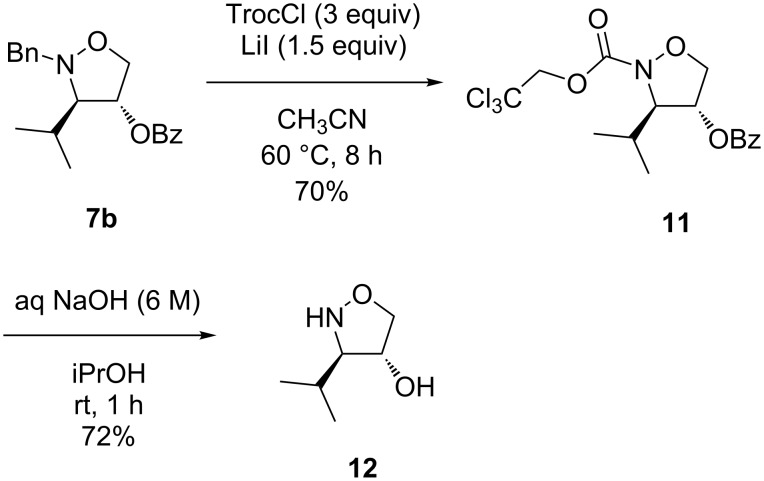
N-debenzylation via *N*-Troc-protected isoxazolidines.

A successful debenzylation was achieved in a two-step procedure using 2,2,2-trichloroethyl chloroformate (TrocCl). However, the protection of the hydroxy group was required first (for the reaction of **8b** with benzoyl chloride, see Supporting Information File 1). The consecutive reaction of the O-benzoylated isoxazolidine **7b** with TrocCl in the presence of lithium iodide [39–40] gave the protected *N*-Troc isoxazolidine **11** in 70% yield (Scheme 5). It is worth mentioning that considering suitable solvents, acetonitrile was superior to 1,2-dichloroethane. Finally, the subsequent basic hydrolysis with 6 M NaOH [39] in isopropyl alcohol [41] to remove the carbamate readily afforded the desired isoxazolidine **12** in good yield (72%). The progress of the reaction was easily followed by TLC (EtOAc), and the N-unsubstituted isoxazolidin-4-ol **12** was identified as a readily oxidizable polar component by permanganate detection even at room temperature. The prior attempts to cleave the Troc group by zinc dust in acetic acid [42] failed due to the reduction of the N–O bond.

## Conclusion

In summary, we have developed a new synthetic approach towards 3-substituted isoxazolidin-4-ols employing the hydroboration–oxidation reaction of 4,5-unsubstituted 2,3-dihydroisoxazoles with basic hydrogen peroxide. A patient addition of both NaOH (aq) and H_2_O_2_ (aq) accompanied by intense cooling was a crucial part of the procedure in terms of product yield. All hydroborations were highly regioselective, leading only to the formation of isoxazolidines with the hydroxy group in the C-4 position. From a stereochemical point of view, the additions took place with an excellent *trans* selectivity with respect to the substituent at C-3.

A sequential oxidation/reduction route was successfully used for the inversion of the C-3/4-*trans* relative configuration of the isoxazolidine ring. The bulkier ʟ-selectride showed an excellent selectivity toward the C-3/4-*cis* isomer compared to lithium borohydride. The final N-debenzylation by a two-step procedure involving a basic hydrolysis of the corresponding *N*-Troc intermediate was successfully exemplified on the 3-isopropyl-substituted derivative. We believe that the presented method will provide a fast and highly stereoselective approach towards both C-3/4-*cis* and C-3/4-*trans* isomers of 3-substituted isoxazolidin-4-ols as valuable structural fragments in drug discovery due to their resemblance with 3-hydroxypyrrolidines.

## Experimental

### Typical procedure for the hydroboration of 2,3-dihydroisoxazoles

**(±)-2-Benzyl-3-phenylisoxazolidin-4-ol (8a):** A round-bottom flask was charged with the 2,3-dihydroisoxazole **5a** (590 mg, 2.49 mmol), evacuated, and flushed with argon. Afterwards, dry THF was added (25 mL), the mixture was cooled to 0 °C, and BH_3_∙THF (5 mL, 5 mmol, 1 M solution in THF) was added dropwise. The reaction mixture was stirred at rt for 12 h. After the disappearance of the starting material (TLC, hexanes/EtOAc, 4:1), a 10% solution of NaOH (7.5 mL) was added dropwise as slowly as possible at 0 °C, followed by a 35% solution of H_2_O_2_ (15 mL) added in a likewise manner. After 3 h of stirring at 0 °C (TLC, hexanes/EtOAc, 1:1), the reaction was diluted with EtOAc (30 mL). The organic layer was separated, washed with brine (2 × 40 mL), dried over MgSO_4_, and concentrated under reduced pressure. The product was purified by FCC (hexanes/EtOAc, 7:3) to give the isoxazolidinol **8a** (485 mg, 1.90 mmol, 76%) as colorless oil. *R*_f_ 0.43 (*n*-hexane/EtOAc, 1:1); IR (ATR) ν_max_: 3392, 3030, 2862, 1495, 1454, 1095, 993, 753, 695, 635, 527 cm^−1^; ^1^H NMR (600 MHz, CDCl_3_) δ 2.44 (bs, 1H, OH), 3.66 (d, *J* = 4.7 Hz, 1H, H-3), 3.81 (dd, *J* = 2.6, 9.3 Hz, 1H, H-5a), 3.82 (d, *J* = 14.2 Hz, 1H, PhCH_2_), 3.98 (d, *J* = 14.2 Hz, 1H, PhCH_2_), 4.11 (dd, *J* = 6.1, 9.3 Hz, 1H, H-5b), 4.48 (ddd, *J* = 2.6, 4.8, 6.1 Hz, 1H, H-4), 7.21–7.43 (m, 10H, H-Ph); ^13^C NMR (150 MHz, CDCl_3_) δ 60.3 (PhCH_2_), 73.8 (C-5), 79.5 (C-3), 83.5 (C-4), 127.4 (CH-Ph), 127.9 (CH-Ph), 128.2 (CH-Ph), 128.3 (CH-Ph), 2×128.9 (CH-Ph), 137.4 (C-Ph), 138.2 (C-Ph); HRMS (ESI) (*m*/*z*): [M + H]^+^ calcd for C_16_H_18_NO_2_, 256.1333; found 256.1329.

### Typical procedure for the oxidation of isoxazolidin-4-ols with Dess–Martin periodinane

**(±)-2-Benzyl-3-phenylisoxazolidin-4-one (9a):** A Schlenk flask was charged with the isoxazolidinol **8a** (450 mg, 1.76 mmol), evacuated, and filled with argon. The starting material was dissolved in anhydrous CH_2_Cl_2_ (18 mL), the reaction mixture was cooled down to 0 °C, and solid Dess–Martin periodinane (1.5 g, 3.54 mmol) was slowly added under a stream of argon. The reaction was stirred at 0 °C for 12 h, and after the complete conversion of the starting material (TLC analysis: hexanes/EtOAc, 1:1), a saturated aq NaHCO_3_ (20 mL) and a saturated aq Na_2_S_2_O_3_∙5H_2_O (20 mL) solution were added. The mixture was stirred for 15 min at 0 °C and then, the solution was allowed to warm to rt. The organic layer was separated and washed with water (2 × 20 mL), dried over MgSO_4_, and evaporated in vacuo. The residue was purified by FCC (hexanes/EtOAc, 9:1) to give the isoxazolidin-4-one **9a** (305 mg, 1.20 mmol, 68%) as yellowish waxy solid that gradually decomposed over time. mp 35–38 °C; *R*_f_ 0.61 (*n*-hexane/EtOAc, 1:1); IR (ATR) ν_max_: 3032, 2871, 2814, 1770, 1495, 1454, 1123, 1048, 737, 695, 615, 545, 470 cm^−1^; ^1^H NMR (300 MHz, CDCl_3_) δ 3.97 (s, 1H, H-3), 3.98 (d, *J* = 14.3 Hz, 1H, PhCH_2_), 4.14 (d, *J* = 15.7 Hz, 1H, H-5a), 4.24 (d, *J* = 14.3 Hz, 1H, PhCH_2_), 4.29 (d, *J* = 15.7, 1H, H-5b), 7.28–7.41 (m, 10H, H-Ph); ^13^C NMR (150 MHz, CDCl_3_) δ 60.7 (PhCH_2_), 70.7 (C-5), 75.1 (C-3), 127.9 (CH-Ph), 128.5 (CH-Ph), 128.7 (CH-Ph), 128.8 (CH-Ph), 129.0 (CH-Ph), 129.3 (CH-Ph), 133.6 (C-Ph), 136.0 (C-Ph), 210.8 (C=O); HRMS (APCI) (*m*/*z*): [M + H]^+^ calcd for C_16_H_16_NO_2_, 254.1176; found, 254.1174.

### Typical procedure for the reduction of isoxazolidin-4-one with ʟ-selectride

**(±)-2-Benzyl-3-phenylisoxazolidin-4-ol (10a):** ʟ-Selectride (1.4 mL, 1.4 mmol, 1 M solution in THF) was added dropwise to a solution of the isoxazolidin-4-one **9a** (280 mg, 1.11 mmol) in anhydrous THF (11 mL) under an argon atmosphere at 0 °C, and the reaction mixture was stirred for 30 min. When TLC analysis showed that the starting isoxazolidinone had disappeared (hexanes/EtOAc, 1:1), a saturated aq NH_4_Cl solution was added slowly (20 mL), and the mixture was stirred for additional 10 min. Afterwards, the mixture was extracted with CH_2_Cl_2_ (3×20 mL). The combined organic layers were washed with water (50 mL), dried over MgSO_4,_ and concentrated under reduced pressure. The product was isolated by FCC (hexanes/EtOAc, 7:3) to give the isoxazolidinol **10a** (215 mg, 0.84 mmol, 76%) as colorless oil. *R*_f_ 0.41 (*n*-hexane/EtOAc, 1:1); IR (ATR) ν_max_: 3421, 3028, 2924, 2868, 1495, 1454, 1107, 1028, 749, 696, 599, 531 cm^−1^; ^1^H NMR (600 MHz, CDCl_3_) δ 1.65 (bs, 1H, OH), 3.70 (d, *J* = 14.4 Hz, 1H, PhCH_2_), 3.79 (d, *J* = 5.5 Hz, 1H, H-3), 3.85 (dd, *J* = 3.5, 9.2 Hz, 1H, H-5a), 4.07 (d, *J* = 14.4 Hz, 1H, PhCH_2_), 4.38 (dd, *J* = 6.1, 9.2 Hz, 1H, H-5b), 4.59 (td, *J* = 3.5, 5.8 Hz, 1H, H-4), 7.24–7.47 (m, 10H, H-Ph); ^13^C NMR (150 MHz, CDCl_3_) δ 60.4 (PhCH_2_), 74.5 (C-5), 75.3 (C-3), 76.8 (C-4), 127.4 (CH-Ph), 128.3 (CH-Ph), 128.4 (CH-Ph), 128.9 (CH-Ph), 129.0 (CH-Ph), 129.2 (CH-Ph), 134.4 (C-Ph), 137.3 (C-Ph); HRMS (ESI) (*m*/*z*): [M + H]^+^ calcd for C_16_H_18_NO_2_, 256.1333; found 256.1329.

### N-Debenzylation with 2,2,2-trichloroethyl chloroformate

**(±)-2,2,2-Trichloroethyl 4-(benzoyloxy)-3-isopropylisoxazolidine-2-carboxylate (11):** 2,2,2-Trichloroethyl chloroformate (0.33 mL, 2.4 mmol) was added dropwise to a stirred solution of the *N*-benzylisoxazolidine **7b** (260 mg, 0.8 mmol) and lithium iodide (160 mg, 1.2 mmol) in anhydrous acetonitrile (4 mL) under argon. The reaction mixture was stirred at 60 °C for 8 h. When the TLC analysis showed that the starting isoxazolidine had disappeared (hexanes/EtOAc, 9:1), a saturated aq NaHCO_3_ (10 mL) solution and CH_2_Cl_2_ (20 mL) were added. After vigorous stirring for additional 5 min, the organic layer was separated, and the aqueous phase was extracted with CH_2_Cl_2_ (10 mL). The combined organic layers were washed with water (20 mL), dried over MgSO_4_, and concentrated under reduced pressure. The product was purified by FCC (hexanes/EtOAc, 9:1) to give the *N*-Troc-isoxazolidine **11** (230 mg, 0.56 mmol, 70%) as colorless sticky oil. *R*_f_ 0.20 (*n*-hexane/EtOAc, 9:1); IR (ATR) ν_max_: 3067, 2963, 2881, 1755, 1717, 1374, 1315, 1265, 1107, 1052, 805, 708, 572 cm^−1^; ^1^H NMR (600 MHz, CDCl_3_): δ 1.09 (d, *J* = 6.7 Hz, 3H, CH_3_), 1.14 (d, *J* = 6.7 Hz, 3H, CH_3_), 1.89–1.97 (m, 1H, *CH*(CH_3_)_2_), 4.12 (dd, *J* = 3.4, 9.5 Hz, 1H, H-5a), 4.22 (d, *J* = 8.4 Hz, 1H, H-3), 4.51 (dd, *J* = 5.9, 9.6 Hz, 1H, H-5b), 4.72 (d, *J* = 11.9 Hz, 1H, Cl_3_CCH_2_O), 4.86 (d, *J* = 11.9 Hz, 1H, Cl_3_CCH_2_O), 5.64 (ddd, *J* = 1.1, 3.5, 5.9 Hz, 1H, H-4), 7.43–7.46 (m, 2H, H-Ph), 7.57–7.60 (m, 1H, H-Ph), 7.95–7.98 (m, 2H, H-Ph); ^13^C NMR (150 MHz, CDCl_3_) δ 19.1 (CH_3_), 19.2 (CH_3_), 29.7 (*C*H(CH_3_)_2_), 72.1 (C-3), 74.4, 75.3 (C-5, CO_2_*C*H_2_), 78.9 (C-4), 94.8 (CCl_3_), 128.6 (CH-Ph), 128.9 (C-Ph), 129.7 (CH-Ph), 133.7 (CH-Ph), 157.1 (*C*O_2_CH_2_), 165.9 (*C*OPh); HRMS (ESI) (*m*/*z*): [M + H]^+^ calcd for C_16_H_19_Cl_3_NO_5_, 410.0324; found 410.0329.

### Hydrolysis of trichloroethyl carbamate

**(±)-3-Isopropylisoxazolidin-4-ol (12):** The NaOH solution (0.6 mL, 3.6 mmol, 6 M) was added to a solution of the *N*-Troc-isoxazolidine **11** (120 mg, 0.29 mmol) in isopropyl alcohol (1.2 mL), and the mixture was stirred at rt for 1 h. After the disappearance of the starting material (TLC, hexanes/EtOAc, 5:1), the solution was neutralized with HCl (6 M). Afterwards, a saturated aq NaHCO_3_ solution (2 mL) and solid NaCl were added, and the resulting slurry was vigorously extracted with EtOAc (3×5 mL). The combined organic layers were dried over MgSO_4_ and concentrated under reduced pressure. The product was purified by FCC (EtOAc) to give the isoxazolidinol **12** (28 mg, 0.21 mmol, 72%) as colorless sticky oil. *R*_f_ 0.25 (EtOAc); IR (ATR): ν_max_ = 3160, 2971, 2899, 2874, 1473, 1093, 1036, 1013, 935, 886, 754, 718, 647 cm^−1^; ^1^H NMR (600 MHz, CDCl_3_) δ 1.02 (d, *J* = 6.7 Hz, 3H, CH_3_), 1.04 (d, *J* = 6.7 Hz, 3H, CH_3_), 1.58–1.66 (m, 1H, *CH*(CH_3_)_2_), 2.90 (dd, *J* = 1.8, 8.8 Hz, 1H, H-3), 3.83 (dd, *J* = 1.9, 9.4 Hz, 1H, H-5a), 3.89 (dd, *J* = 5.0, 9.4 Hz, 1H, H-5b), 4.51 (dt, *J* = 2.0, 5.0 Hz, 1H, H-4); ^13^C NMR (150 MHz, CDCl_3_) δ 19.8 (CH_3_), 20.0 (CH_3_), 29.1 (*C*H(CH_3_)_2_), 75.7 (C-3), 78.0 (C-5), 78.4 (C-4); HRMS (ESI) (*m*/*z*): [M + H]^+^ calcd for C_6_H_14_NO_2_, 132.1020; found 132.1020.

## Supporting Information

File 1 Detailed experimental procedures, analytical data, and NMR spectra of all compounds.
