# Supplementary material for: Synthesis of 3-substituted isoxazolidin-4-ols using hydroboration–oxidation reactions of 4,5-unsubstituted 2,3-dihydroisoxazoles
Source: Beilstein J Org Chem. 2020 Jun 16;16:1313–9. doi: 10.3762/bjoc.16.112 (PMC7308612; doi:10.3762/bjoc.16.112)
Supplement: File 1 — Detailed experimental procedures, analytical data, and NMR spectra of all compounds. [file Beilstein_J_Org_Chem-16-1313-s001.pdf]

BEILSTEIN JOURNAL OF ORGANIC CHEMISTRY

Supporting Information

for

Synthesis of 3-substituted isoxazolidin-4-ols using hydroboration–oxidation reactions of 4,5-unsubstituted 2,3-dihydroisoxazoles

Lívia Dikošová, Júlia Laceková, Ondrej Záborský and Róbert Fischer

Beilstein J. Org. Chem. **2020,** *16,* 1313–1319. doi:10.3762/bjoc.16.112

Detailed experimental procedures, analytical data, and NMR spectra of all compounds

License and Terms: This is a supporting information file under the terms of the Creative Commons Attribution License (http://creativecommons.org/licenses/by/4.0). Please note that the reuse, redistribution and reproduction in particular requires that the authors and source are credited.
The license is subject to the *Beilstein Journal of Organic Chemistry* terms and conditions: (https://www.beilstein-journals.org/bjoc)

Table of contents

1.	Experimental section	S2–S10
2.	^{1}H and ^{13}C NMR spectra of compound **7a**	S11
3.	^{1}H and ^{13}C NMR spectra of compound **7b**	S12
4.	^{1}H and ^{13}C NMR spectra of compound **8a**	S13
5.	^{1}H and ^{13}C NMR spectra of compound **8b**	S14
6.	^{1}H and ^{13}C NMR spectra of compound **8c**	S15
7.	^{1}H and ^{13}C NMR spectra of compound **9a**	S16
8.	^{1}H and ^{13}C NMR spectra of compound **9b**	S17
9.	^{1}H and ^{13}C NMR spectra of compound **9c**	S18
10.	^{1}H and ^{13}C NMR spectra of compound **10a**	S19
11.	^{1}H and ^{13}C NMR spectra of compound **10b**	S20
12.	^{1}H and ^{13}C NMR spectra of compound **10c**	S21
13.	^{1}H and ^{13}C NMR spectra of compound **11**	S22
14.	^{1}H and ^{13}C NMR spectra of compound **12**	S23
15.	References	S24

Experimental cection

General

Flash column chromatography (FCC) was carried out with a Büchi system (Pump Manager C-615 and Fraction Collector C-660) using Normasil 60 silica gel (0.040–0.063 mm; VWR). Thin-layer chromatography (TLC) analysis was carried out using TLC silica gel 60 F_{254} (aluminium sheets, Merck), and plates were visualized with UV light or by treatment with permanganate solution followed by heating. Optical rotations were measured with a JASCO P-2000 digital polarimeter with a Na-D lamp (10 cm cell length). Concentrations (*c*) are given in gram per 100 mL. Infrared (IR) spectra were recorded as neat samples with a Nicolet 5700 FTIR spectrometer with an ATR Smart Orbit Diamond adapter (Thermo Electron Corporation). NMR spectra were recorded with a Varian INOVA-300 spectrometer (^{1}H, 299.95 MHz, and ^{13}C, 75.42 MHz) and a Varian VNMRS-600 instrument (^{1}H, 599.75 MHz, and ^{13}C, 150.81 MHz) in $CDCl_3$ using tetramethylsilane as the internal standard. Data are presented as follows: chemical shift (in ppm), multiplicity (s = singlet, d = doublet, t = triplet, q = quartet, dd = doublet of doublets, ddd = doublet of doublet of doublets, td = triplet of doublets, dt = doublet of triplets, m =multiplet, bs = broad singlet), coupling constants (*J*/Hz) and integration. HRMS analysis was carried out with an Orbitrap Velos Pro spectrometer (Thermo Fisher Scientific). All solvents used were dried and distilled according to conventional methods. 2,3-Dihydroisoxazoles **5a**, **5b** and benzoylated isoxazolidine-4,5-diols **6a**, **6b** were prepared using already published procedures [1,2].

(±)-2-Benzyl-3-phenylisoxazolidin-4-yl benzoate (7a)

A round-bottom reaction flask was charged with isoxazolidinyl dibenzoate **6a** (350 mg, 0.73 mmol), sealed with a rubber septum, evacuated, and filled with argon. Anhydrous CH_2Cl_2 was added (0.7 mL) followed by Et_3SiH (350 µL, 2.19 mmol), and the resulting solution was cooled in an ice-water bath (0 °C). TMSOTf (270 µL, 1.49 mmol) was added dropwise to the stirred solution. After stirring at 0 °C for 5 min, the cooling bath was removed, and the mixture was further stirred at room temperature for 2 h. After this time, TLC showed that the reaction was complete (hexanes/ethyl acetate, 9:1). The reaction mixture was cooled in an ice-water bath and the reaction was quenched by addition of sat. aq $NaHCO_3$ solution (5 mL). Afterwards, the mixture was diluted with water (5 mL) and extracted with CH_2Cl_2 (2 × 5 mL). The combined organic layers were washed with water (10 mL), dried with $MgSO_4$ and evaporated in vacuo. The product was isolated by FCC (hexanes/EtOAc, 9:1) to give the isoxazolidine **7a** (195 mg, 0.54 mmol, 74%) as a white solid. mp 78–80 °C; R_f = 0.24 (*n*-hexane/EtOAc, 9:1); IR (ATR): υ_{max} = 3030, 2873, 1713, 1448, 1271, 1112, 1071, 979, 712, 694, 633, 536 cm^{-1}; ^{1}H NMR (600 MHz, $CDCl_3$) δ 3.96 (d, *J* = 14.1 Hz, 1H, $PhCH_2$), 4.07–4.11 (m, 3H, H-3, H-5a, $PhCH_2$), 4.37 (dd, *J* = 5.8, 10.3 Hz, 1H, H-5b), 5.58 (ddd, *J* = 2.0, 4.3, 5.8 Hz, 1H, H-4), 7.23–7.60 (m, 13H, H-Ph), 8.06–8.08 (m, 2H, H-Ph); ^{13}C NMR (150 MHz, $CDCl_3$) δ 60.3 ($PhCH_2$),

72.1 (C-5), 75.8 (C-3), 85.6 (C-4), 127.5 (CH-Ph), 128.0 (CH-Ph), 128.3 (CH-Ph), 128.4 (CH-Ph), 128.6 (CH-Ph), 2 × 128.9 (CH-Ph), 129.7 (C-Ph), 129.9 (CH-Ph), 133.5 (CH-Ph), 137.4 (C-Ph), 137.7 (C-Ph), 166.3 (C=O); HRMS (ESI): calcd. for $C_{23}H_{22}NO_3$ $[M+H]^+$ 360.1595; found 360.1593.

(±)-2-Benzyl-3-isopropylisoxazolidin-4-yl benzoate (7b)

A round-bottom reaction flask was charged with isoxazolidinyl dibenzoate **6b** (360 mg, 0.81 mmol), sealed with a rubber septum, evacuated, and filled with argon. Anhydrous CH_2Cl_2 was added (0.8 mL) followed by Et_3SiH (390 µL, 2.44 mmol), and the resulting solution was cooled in an ice-water bath (0 °C). TMSOTf (290 µL, 1.60 mmol) was added dropwise to the stirred solution. After stirring at 0 °C for 5 min, the cooling bath was removed, and the mixture was further stirred at room temperature for 2 h. After this time, TLC showed that the reaction was complete (hexanes/ethyl acetate, 4:1). The reaction mixture was cooled in an ice-water bath and the reaction was quenched by addition of saturated aqueous solution of $NaHCO_3$ (5 mL). Afterwards, the mixture was diluted with water (5 mL) and extracted with CH_2Cl_2 (2 × 5 mL). The combined organic layers were washed with water (10 mL), dried with $MgSO_4$ and evaporated in vacuo. The product was isolated by FCC (hexanes/EtOAc, 9:1) to give the isoxazolidine **7b** (210 mg, 0.65 mmol, 80%) as a colourless oil. R_f = 0.22 (*n*-hexane/EtOAc, 9:1); IR (ATR): υ_{max} = 2958, 2873, 1716, 1452, 1270, 1109, 1069, 1026, 709, 697 cm^{-1}; ^1H NMR (600 MHz, $CDCl_3$) δ 1.00 (d, *J* = 6.8 Hz, 3H, CH_3), 1.02 (d, *J* = 6.8 Hz, 3H, CH_3), 1.80–1.88 [m, 1H, $\underline{CH}(CH_3)_2$], 3.01 (dd, *J* = 2.3, 6.6 Hz, 1H, H-3), 4.10 (dd, *J* = 2.5, 10.3 Hz, 1H, H-5a), 4.11 (d, *J* = 13.3 Hz, 1H, $PhCH_2$), 4.16 (d, *J* = 13.3 Hz, 1H, $PhCH_2$), 4.19 (dd, *J* = 5.7, 10.3 Hz, 1H, H-5b), 5.61 (dt, *J* = 2.4, 2.5, 5.7 Hz, 1H, H-4), 7.25–7.62 (m, 8H, H-Ph), 8.06–8.08 (m, 2H, H-Ph); ^{13}C NMR (150 MHz, $CDCl_3$) δ 18.8 (CH_3), 19.9 (CH_3), 29.7 [$\underline{C}H(CH_3)_2$], 61.6 ($PhCH_2$), 72.0 (C-5), 76.2 (C-3), 80.8 (C-4), 127.5 (CH-Ph), 128.5 (CH-Ph), 128.7 (CH-Ph), 129.2 (CH-Ph), 129.8 (CH-Ph), 129.9 (C-Ph), 133.5 (CH-Ph), 137.6 (C-Ph), 166.2 (C=O); HRMS (ESI): calcd. for $C_{20}H_{24}NO_3$ $[M+H]^+$ 326.1751; found 326.1750.

From 8b by reaction with benzoyl chloride

Isoxazolidin-4-ol **8b** (270 mg, 1.22 mmol) was dissolved in CH_2Cl_2 (6 mL), and then benzoyl chloride (0.29 mL, 2.50 mmol), pyridine (0.3 mL, 3.68 mmol) and DMAP (29 mg, 0.24 mmol) were added. The reaction mixture was stirred at rt overnight. After the reaction was complete (TLC; hexanes/EtOAc, 7:3), above mixture was diluted with water (15 mL) and repeatedly extracted with CH_2Cl_2 (3 × 15 mL). The combined organic layers were dried over $MgSO_4$ and the solvent was removed by rotary evaporation. The product was isolated by FCC (CH_2Cl_2) to give the isoxazolidine **7b** (320 mg, 0.98 mmol, 80%) as a colourless oil; R_f = 0.18 (CH_2Cl_2). All analytical data were consistent with those described above.

(±)-2-Benzyl-3-phenylisoxazolidin-4-ol (8a)

A round-bottom flask was charged with 2,3-dihydroisoxazole **5a** (590 mg, 2.49 mmol), evacuated and flushed with argon. Afterwards, dry THF was added (25 mL), the mixture was cooled to 0 °C and BH_3·THF (5 mL, 5 mmol, 1 M solution in THF) was added dropwise. The reaction mixture was stirred at rt for 12 h. After the disappearance of the starting material (TLC, hexanes/EtOAc, 4:1), a 10% solution of NaOH (7.5 mL) was added dropwise as slowly as possible at 0 °C, followed by a 35% solution of H_2O_2 (15 mL) added in likewise manner. After 3 h of stirring at 0 °C (TLC, hexanes/EtOAc, 1:1), the reaction was diluted with EtOAc (30 mL). The organic layer was separated, washed with brine (2 × 40 mL), dried over $MgSO_4$ and concentrated under reduced pressure. The product was purified by FCC (hexanes/EtOAc, 7:3) to give isoxazolidinol **8a** (485 mg, 1.90 mmol, 76%) as a colourless oil. R_f = 0.43 (*n*-hexane/EtOAc, 1:1); IR (ATR): υ_{max} = 3392, 3030, 2862, 1495, 1454, 1095, 993, 753, 695, 635, 527 cm^{-1}; ^1H NMR (600 MHz, $CDCl_3$) δ 2.44 (bs, 1H, OH), 3.66 (d, *J* = 4.7 Hz, 1H, H-3), 3.81 (dd, *J* = 2.6, 9.3 Hz, 1H, H-5a), 3.82 (d, *J* = 14.2 Hz, 1H, $PhCH_2$), 3.98 (d, *J* = 14.2 Hz, 1H, $PhCH_2$), 4.11 (dd, *J* = 6.1, 9.3 Hz, 1H, H-5b), 4.48 (ddd, *J* = 2.6, 4.8, 6.1 Hz, 1H, H-4), 7.21–7.43 (m, 10H, H-Ph); ^{13}C NMR (150 MHz, $CDCl_3$) δ 60.3 ($PhCH_2$), 73.8 (C-5), 79.5 (C-3), 83.5 (C-4), 127.4 (CH-Ph), 127.9 (CH-Ph), 128.2 (CH-Ph), 128.3 (CH-Ph), 2 × 128.9 (CH-Ph), 137.4 (C-Ph), 138.2 (C-Ph); HRMS (ESI): calcd. for $C_{16}H_{18}NO_2$ $[M+H]^+$ 256.1333; found 256.1329.

From 7a by hydrolysis with K_2CO_3 in aqueous methanol

Isoxazolidinyl benzoate **7a** (200 mg; 0.56 mmol) was dissolved in aqueous methanol (MeOH/H_2O,10:1; 5.5 mL), potassium carbonate (39 mg; 0.28 mmol) was added and the solution was stirred at rt for 12 h. When TLC showed that the starting isoxazolidine had disappeared (TLC; hexanes/EtOAc, 1:1), water (10 mL) and Et_2O (10 ml) were added. After stirring for additional 5 minutes, the organic layer was separated, and the aqueous phase was extracted with Et_2O (2 × 10 mL). The combined organic layers were dried over $MgSO_4$ and concentrated under reduced pressure. The product was purified by FCC (hexanes/EtOAc, 7:3) to give isoxazolidinol **8a** (125 mg; 0.49 mmol; 88%) as a colourless oil. All analytical data were consistent with those described above.

(±)-2-Benzyl-3-isopropylisoxazolidin-4-ol (8b)

A round-bottom flask was charged with 2,3-dihydroisoxazole **5b** (1 g; 4.92 mmol), evacuated and flushed with argon. Afterwards, dry THF was added (50 mL), the mixture was cooled to 0 °C and BH_3·THF (10 mL; 10 mmol; 1 M solution in THF) was added dropwise. The reaction mixture was stirred at rt for 12 h. After the disappearance of the starting material (TLC, hexanes/EtOAc, 95:5) a 10% solution of NaOH (15 mL) was added dropwise as slowly as possible at 0 °C, followed by a 35% solution of H_2O_2 (30 mL) added in likewise manner. After 3 h of stirring at 0 °C (TLC; hexanes/EtOAc, 1:1) the reaction was diluted with EtOAc (50 mL). The organic layer was separated, washed with brine (2 × 40 mL), dried over $MgSO_4$ and concentrated under reduced pressure. The

product was purified by FCC (hexanes/EtOAc, 7:3) to give isoxazolidinol **8b** (1 g; 4.52 mmol, 92%) as a colourless waxy solid. mp 32–34 °C; R_f = 0.37 (*n*-hexane/EtOAc, 1:1); IR (ATR): υ_{max} = 3300, 2961, 2873, 1455, 1370, 1083, 1001, 957, 758, 700, 639 cm^{-1}; ^{1}H NMR (600 MHz, $CDCl_3$) δ 0.97 (d, *J* = 6.8 Hz, 3H, CH_3), 1.00 (d, *J* = 6.8 Hz, 3H, CH_3), 1.68–1.75 [m, 1H, $\underline{CH}(CH_3)_2$], 1.97 (bs, 1H, OH), 2.62 (dd, *J* = 2.4, 6.6 Hz, 1H, H-3), 3.86–3.89 (m, 2H, H-5a, H-5b), 4.05 (d, *J* = 13.7 Hz, 1H, $PhCH_2$), 4.10 (d, *J* = 13.7 Hz, 1H, $PhCH_2$), 4.43–4.45 (m, 1H, H-4), 7.25–7.40 (m, 5H, H-Ph); ^{13}C NMR (150 MHz, $CDCl_3$): δ 18.7 (CH_3), 20.0 (CH_3), 29.7 [$\underline{C}H(CH_3)_2$], 61.9 ($PhCH_2$), 73.9 (C-5), 78.0 (C-3), 79.9 (C-4), 127.4 (CH-Ph), 128.4 (CH-Ph), 129.1 (CH-Ph), 137.8 (C-Ph); HRMS (ESI): calcd. for $C_{13}H_{20}NO_2$ $[M+H]^+$ 222.1489; found 222.1491.

From 7b by hydrolysis with K_2CO_3 in aqueous methanol

Isoxazolidinyl benzoate **7b** (100 mg; 0.31 mmol) was dissolved in aqueous methanol ($MeOH/H_2O$,10:1; 3 mL), potassium carbonate (22 mg; 0.16 mmol) was added and the solution was stirred at rt for 12 h. When TLC showed that the starting isoxazolidine had disappeared (TLC; hexanes/EtOAc, 1:1), water (5 mL) and Et_2O (5 ml) were added. After stirring for additional 5 minutes, the organic layer was separated, and the aqueous phase was extracted with Et_2O (2 × 5 mL). The combined organic layers were dried over $MgSO_4$ and concentrated under reduced pressure. The product was purified by FCC (hexanes/EtOAc, 7:3) to give isoxazolidinol **8b** (60 mg; 0.27 mmol; 87%) as a colourless waxy solid. All analytical data were consistent with those described above.

(3*S*,4*R*)-2-Benzyl-3-[(*S*)-2,2-dimethyl-1,3-dioxolan-4-yl]isoxazolidin-4-ol (8c)

A round-bottom flask was charged with 2,3-dihydroisoxazole **5c** (920 mg; 3.52 mmol), evacuated and flushed with argon. Afterwards, dry THF was added (35 mL), the mixture was cooled to 0 °C and BH_3·THF (7 mL; 7 mmol; 1 M solution in THF) was added dropwise. The reaction mixture was stirred at rt for 12 h. After the disappearance of the starting material (TLC, hexanes/EtOAc, 9:1) a 10% solution of NaOH (11 mL) was added dropwise as slowly as possible at 0 °C, followed by a 35% solution of H_2O_2 (21 mL) added in likewise manner. After 3 h of stirring at 0 °C (TLC; hexanes/EtOAc, 7:3) the reaction was diluted with EtOAc (30 mL). The organic layer was separated, washed with brine (2 × 40 mL), dried over $MgSO_4$ and concentrated under reduced pressure. The product was purified by FCC (hexanes/EtOAc, 1:1) to give isoxazolidinol **8c** (645 mg; 2.31 mmol; 65%) as a colourless waxy solid. mp 53–55 °C; R_f = 0.28 (*n*-hexane/EtOAc, = 1:1); $[\alpha]_D^{25}$ = +29.25 (*c* =1.01, $CHCl_3$); IR (ATR): υ_{max} = 3290, 2987, 2887, 1368, 1264, 1204, 1150, 1073, 1039, 1002, 840, 741, 701, 513 cm^{-1}; ^{1}H NMR (600 MHz, $CDCl_3$) δ 1.31 (s, 3H, CH_3), 1.37 (s, 3H, CH_3), 2.29 (bs, 1H, OH), 3.06 (dd, *J* = 1.5, 8.8 Hz, 1H, H-3), 3.51 (dd, *J* = 5.8, 8.5 Hz, 1H, H-2´a), 3.93 (dt, *J* = 6.0, 8.8 Hz, 1H, H-1´), 4.01 (dd, *J* = 6.2, 8.5 Hz, 1H, H-2´b), 4.04 (dd, *J* = 3.7, 9.4 Hz, 1H, H-5a), 4.12 (d, *J* = 12.8 Hz, 1H, $PhCH_2$), 4.15 (dd, *J* = 6.1, 9.4 Hz, 1H, H-5b), 4.25 (d, *J* = 12.8 Hz, 1H,

$PhCH_2$), 4.81–4.84 (m, 1H, H-4), 7.27–7.39 (m, 5H, H-Ph); ^{13}C NMR (150 MHz, $CDCl_3$) δ 25.4 (CH_3), 27.0 (CH_3), 61.6 ($PhCH_2$), 68.0 (C-2'), 73.5 (C-5), 75.3, 75.5 (C-3, C-1'), 78.6 (C-4), 109.6 [$\underline{C}(CH_3)_2$], 127.8 (CH-Ph), 128.6 (CH-Ph), 129.6 (CH-Ph), 136.9 (C-Ph); HRMS (ESI): calcd. for $C_{15}H_{22}NO_4$ $[M+H]^+$ 280.1544; found 280.1545.

(±)-2-Benzyl-3-phenylisoxazolidin-4-one (9a)

A Schlenk flask was charged with isoxazolidinol **8a** (450 mg, 1.76 mmol), evacuated and filled with argon. The starting material was dissolved in anhydrous CH_2Cl_2 (18 mL), the reaction mixture was cooled to 0 °C and solid Dess–Martin periodinane (1.5 g, 3.54 mmol) was slowly added under stream of argon. The reaction was stirred at 0 °C for 12 h and after complete conversion of the starting material (TLC, hexanes/EtOAc, 1:1), a sat. aq $NaHCO_3$ solution (20 mL) and a sat. aq $Na_2S_2O_3{\cdot}5H_2O$ solution (20 mL) were added. The mixture was stirred for 15 min at 0 °C, and then the solution was allowed to warm to rt. The organic layer was separated and washed with water (2 × 20 mL), dried over $MgSO_4$ and evaporated in vacuo. The residue was purified by FCC (hexanes/EtOAc, 9:1) to give isoxazolidin-4-one **9a** (305 mg, 1.20 mmol, 68%) as a yellowish waxy solid that gradually decomposed over time. mp 35–38 °C; R_f = 0.61 (*n*-hexane/EtOAc, 1:1); IR (ATR): υ_{max} = 3032, 2871, 2814, 1770, 1495, 1454, 1123, 1048, 737, 695, 615, 545, 470 cm^{-1}; ^{1}H NMR (300 MHz, $CDCl_3$) δ 3.97 (s, 1H, H-3), 3.98 (d, *J* = 14.3 Hz, 1H, $PhCH_2$), 4.14 (d, *J* = 15.7 Hz, 1H, H-5a), 4.24 (d, *J* = 14.3 Hz, 1H, $PhCH_2$), 4.29 (d, *J* = 15.7, 1H, H-5b), 7.28–7.41 (m, 10H, H-Ph); ^{13}C NMR (150 MHz, $CDCl_3$) δ 60.7 ($PhCH_2$), 70.7 (C-5), 75.1 (C-3), 127.9 (CH-Ph), 128.5 (CH-Ph), 128.7 (CH-Ph), 128.8 (CH-Ph), 129.0 (CH-Ph), 129.3 (CH-Ph), 133.6 (C-Ph), 136.0 (C-Ph), 210.8 (C=O); HRMS (APCI): calcd. for $C_{16}H_{16}NO_2$ $[M+H]^+$ 254.1176; found 254.1174.

(±)-2-Benzyl-3-isopropylisoxazolidin-4-one (9b)

A Schlenk flask was charged with isoxazolidinol **8b** (500 mg; 2.26 mmol), evacuated and filled with argon. The starting material was dissolved in anhydrous CH_2Cl_2 (23 mL), the reaction mixture was cooled to 0 °C and solid Dess–Martin periodinane (1.9 g; 4.48 mmol) was slowly added under stream of argon. The reaction was stirred at 0 °C for 12 h and after complete conversion of the starting material (TLC, hexanes/EtOAc, 7:3), a sat. aq $NaHCO_3$ solution (25 mL) and a sat. aq $Na_2S_2O_3{\cdot}5H_2O$ solution (25 mL) were added. The mixture was stirred for 15 min at 0 °C, and then the solution was allowed to warm to rt. The organic layer was separated and washed with water (2 × 20 mL), dried over $MgSO_4$ and evaporated in vacuo. The residue was purified by FCC (hexanes/EtOAc, 9:1) to give isoxazolidin-4-one **9b** (320 mg; 1.46 mmol; 65%) as a yellowish waxy solid that gradually decomposed over time. mp 38–40 °C; R_f = 0.67 (*n*-hexane/EtOAc, 1:1); IR (ATR): υ_{max} = 2966, 2872, 2810, 1754, 1451, 1367, 1051, 753, 701, 641, 484 cm^{-1}; ^{1}H NMR (600 MHz, $CDCl_3$) δ 0.98 (d, *J* = 6.9 Hz, 3H, CH_3), 1.06 (d, *J* = 6.9 Hz, 3H, CH_3), 1.95–2.10 [m, 1H, $\underline{CH}(CH_3)_2$], 2.84 (dd, *J* = 0.8, 4.8 Hz, 1H, H-3), 3.88 (d, *J* = 15.9 Hz, 1H, H-5a), 4.03 (d, *J* = 13.5 Hz, 1H, $PhCH_2$), 4.11 (dd, *J*

= 0.8, 15.9 Hz, 1H, H-5b), 4.12 (d, J = 13.5 Hz, 1H, $PhCH_2$), 7.27–7.41 (m, 5H, H-Ph); ^{13}C NMR (150 MHz, $CDCl_3$) δ 18.7 (CH_3), 18.8 (CH_3), 29.4 [<u>C</u>H(CH_3)$_2$], 62.1 ($PhCH_2$), 68.7 (C-5), 74.1 (C-3), 127.9 (CH-Ph), 128.6 (CH-Ph), 129.2 (CH-Ph), 136.3 (C-Ph), 215.0 (C=O); HRMS (APCI): calcd. for $C_{13}H_{18}NO_2$ $[M+H]^+$ 220.1333; found 220.1336.

(*R*)-2-Benzyl-3-[(*S*)-2,2-dimethyl-1,3-dioxolan-4-yl]isoxazolidin-4-one (9c)

A Schlenk flask was charged with isoxazolidinol **8c** (615 mg; 2.20 mmol), evacuated and filled with argon. The starting material was dissolved in anhydrous CH_2Cl_2 (22 mL), the reaction mixture was cooled to 0 °C and solid Dess–Martin periodinane (1.8 g; 4.4 mmol) was added slowly under stream of argon. The reaction was stirred at 0 °C for 12 h and after complete conversion of the starting material (TLC, hexanes/EtOAc, 1:1), a sat. aq $NaHCO_3$ solution (20 mL) and a sat. aq $Na_2S_2O_3 \cdot 5H_2O$ solution (20 mL) were added. The mixture was stirred for 15 min at 0 °C, and then the solution was allowed to warm to rt. The organic layer was separated and washed with water (2 × 20 mL), dried over $MgSO_4$ and evaporated in vacuo. The product was purified by FCC (hexanes/EtOAc, 4:1) isoxazolidin-4-one **9c** (390 mg; 1.41 mmol, 64%) as a yellowish waxy solid that gradually decomposed over time; mp 34–36 °C. R_f = 0.59 (*n*-hexane/EtOAc, 1:1); $[\alpha]_D^{25}$ = –45.07 (*c* = 1.02, $CHCl_3$); IR (ATR): υ_{max} = 2987, 2891, 2827, 1765, 1370, 1262, 1202, 1157, 1046, 841, 758, 701, 636, 576, 522 cm^{-1}; ^{1}H NMR (600 MHz, $CDCl_3$) δ 1.36 (s, 3H, CH_3), 1.43 (s, 3H, CH_3), 3.37 (d, J = 3.8 Hz, 1H, H-3), 3.92 (d, J = 16.0 Hz, 1H, H-5a), 4.00 (dd, J = 6.5, 8.1 Hz, 1H, H-2´a), 4.03 (dd, J = 7.0, 8.1 Hz, 1H, H-2´b), 4.10 (d, J = 13.9 Hz, 1H, $PhCH_2$), 4.13 (d, J = 16.0 Hz, 1H, H-5b), 4.35 (d, J = 13.9 Hz, 1H, $PhCH_2$), 4.50 (dt, J = 3.8, 6.8 Hz, 1H, H-1´), 7.30–7.42 (m, 5H, H-Ph); ^{13}C NMR (150 MHz, $CDCl_3$) δ 25.1 (CH_3), 26.4 (CH_3), 62.5 ($PhCH_2$), 65.2 (C-2'), 2 × 69.1 (C-3, C-5), 75.0 (C-1'), 109.9 [<u>C</u>(CH_3)$_2$], 128.0 (CH-Ph), 128.6 (CH-Ph), 129.4 (CH-Ph), 135.9 (C-Ph), 211.5 (C=O); HRMS (APCI): calcd. for $C_{15}H_{20}NO_4$ $[M+H]^+$ 278.1387; found 278.1377.

(±)-2-Benzyl-3-phenylisoxazolidin-4-ol (10a)

L-Selectride (1.4 mL, 1.4 mmol, 1 M solution in THF) was added dropwise to a solution of isoxazolidin-4-one **9a** (280 mg, 1.11 mmol) in anhydrous THF (11 mL) under argon at 0 °C, and the reaction mixture was stirred for 30 min. When TLC showed that the starting isoxazolidinone disappeared (TLC; hexanes/EtOAc, 1:1), a sat. aq NH_4Cl was added slowly (20 mL) and the mixture was stirred for additional 10 min. Afterwards, the mixture was extracted with CH_2Cl_2 (3 × 20 mL). The combined organic layers were washed with water (50 mL), dried over $MgSO_4$ and concentrated under reduced pressure. The product was isolated by FCC (hexanes/EtOAc, 7:3) to give isoxazolidinol **10a** (215 mg, 0.84 mmol, 76%) as a colourless oil. R_f = 0.41 (*n*-hexane/EtOAc, 1:1); IR (ATR): υ_{max} = 3421, 3028, 2924, 2868, 1495, 1454, 1107, 1028, 749, 696, 599, 531 cm^{-1}; ^{1}H NMR (600 MHz, $CDCl_3$) δ 1.65 (bs, 1H, OH), 3.70 (d, J = 14.4 Hz, 1H, $PhCH_2$), 3.79 (d, J = 5.5 Hz, 1H, H-3), 3.85 (dd, J = 3.5, 9.2 Hz, 1H, H-5a), 4.07 (d, J = 14.4 Hz, 1H, $PhCH_2$), 4.38 (dd, J = 6.1, 9.2 Hz,

1H, H-5b), 4.59 (td, J = 3.5, 5.8 Hz, 1H, H-4), 7.24–7.47 (m, 10H, H-Ph); ^{13}C NMR (150 MHz, $CDCl_3$) δ 60.4 ($PhCH_2$), 74.5 (C-5), 75.3 (C-3), 76.8 (C-4), 127.4 (CH-Ph), 128.3 (CH-Ph), 128.4 (CH-Ph), 128.9 (CH-Ph), 129.0 (CH-Ph), 129.2 (CH-Ph), 134.4 (C-Ph), 137.3 (C-Ph); HRMS (ESI): calcd. for $C_{16}H_{18}NO_2$ $[M+H]^+$ 256.1333; found 256.1329.

(±)-2-Benzyl-3-isopropylisoxazolidin-4-ol (10b)

L-Selectride (1.8 mL; 1.8 mmol; 1 M solution in THF) was added dropwise to a solution of isoxazolidin-4-one **9b** (300 mg, 1.37 mmol) in anhydrous THF (14 mL) under argon at 0 °C, and the reaction mixture was stirred for 30 min. When TLC showed that the starting isoxazolidinone disappeared (TLC; hexanes/EtOAc, 1:1), a sat. aq NH_4Cl was added slowly (20 mL) and the mixture was stirred for additional 10 min. Afterwards, it was extracted with CH_2Cl_2 (3 × 20 mL). The combined organic layers were washed with water (50 mL), dried over $MgSO_4$ and concentrated under reduced pressure. The product was purified by FCC (hexanes/EtOAc, 7:3) to give isoxazolidinol **10b** (155 mg; 0.70 mmol; 51%) as a yellowish waxy solid. mp 46–48 °C; R_f = 0.33 (*n*-hexane/EtOAc, 1:1); IR (ATR): υ_{max} = 3358, 2968, 2866, 1454, 1362, 1209, 1026, 944, 879, 801, 745, 696, 602, 509 cm^{-1}; ^{1}H NMR (300 MHz, $CDCl_3$) δ 1.06 (d, J = 6.5 Hz, 3H, CH_3), 1.12 (d, J = 6.5 Hz, 3H, CH_3), 2 .05–2.17 [m, 1H, <u>CH</u>(CH_3)$_2$], 2.31 (bs, 1H, OH), 2.57 (dd, J = 3.5, 8.9 Hz, 1H, H-3), 3.82 (d, J = 9.5 Hz, 1H, H-5a), 3.89 (s, 2H, $PhCH_2$), 4.02 (dd, J = 2.6, 9,5 Hz, 1H, H-5b), 4.49–4.58 (m, 1H, H-4), 7.23–7.42 (m, 5H, H-Ph); ^{13}C NMR (150 MHz, $CDCl_3$) δ 20.4 (CH_3), 21.1 (CH_3), 28.2 [<u>C</u>H(CH_3)$_2$], 62.9 ($PhCH_2$), 71.6 (C-5), 76.9 (C-3), 127.3 (CH-Ph), 128.4 (CH-Ph), 128.9 (CH-Ph), 138.1 (C-Ph). The signal for the C-4 carbon atom is missing, however, NMR spectroscopic data nearly correspond with those for **10a** and **10c**; HRMS (ESI): calcd. for $C_{13}H_{20}NO_2$ $[M+H]^+$ 222.1489; found 222.1491.

(3*S*,4*S*)-2-Benzyl-3-[(*S*)-2,2-dimethyl-1,3-dioxolan-4-yl]isoxazolidin-4-ol (10c)

L-Selectride (1.7 mL; 1.7 mmol; 1 M solution in THF) was added dropwise to a solution of isoxazolidin-4-one **9c** (360 mg; 1.30 mmol) in anhydrous THF (13 mL) under argon at 0 °C, and the reaction mixture was stirred for 30 min. When TLC showed that the starting isoxazolidinone disappeared (TLC; hexanes/EtOAc, 1:1), a sat. aq NH_4Cl was added slowly (20 mL) and the mixture was stirred for additional 10 min. Afterwards, it was extracted with CH_2Cl_2 (3 × 20 mL). The combined organic layers were washed with water (50 mL), dried over $MgSO_4$ and concentrated under reduced pressure. The product was purified by FCC (hexanes/EtOAc, 1:1) to give isoxazolidinol **10c** (260 mg; 0.93 mmol; 72%) as a colourless waxy solid. mp 88–90 °C; R_f = 0.24 (*n*-hexane/EtOAc, 1:1); $[\alpha]_D^{25}$ = –65.53 (c = 1.00, $CHCl_3$); IR (ATR): υ_{max} = 3464, 2989, 2918, 1452, 1373, 1264, 1203, 1158, 1017, 848, 746, 699, 630, 518 cm^{-1}; ^{1}H NMR (600 MHz, $CDCl_3$) δ 1.34 (s, 3H, CH_3), 1.39 (s, 3H, CH_3), 2.87 (bs, 1H, OH), 3.16 (dd, J = 6.0, 7.9 Hz, 1H, H-3), 3.56 (dd, J = 6.4, 8.5 Hz, 1H, H-2´a), 3.87 (dd, J = 2.0, 9.7 Hz, 1H, H-5a), 3.89 (d, J = 13.7 Hz, 1H, $PhCH_2$), 3.99 (d, J = 13.6 Hz, 1H, $PhCH_2$), 4.01 (dd, J = 6.1, 8.5 Hz, 1H, H-2´b), 4.18 (dd, J = 4.8, 9.6 Hz, 1H, H-5b), 4.42 (dt, J = 6.3,

7.9 Hz, 1H, H-1´), 4.82–4.87 (m, 1H, H-4), 7.27–7.36 (m, 5H, H-Ph); ^{13}C NMR (150 MHz, $CDCl_3$) δ 25.4 (CH_3), 26.9 (CH_3), 61.9 (CH_2Ph), 67.8 (C-2'), 70.9 (C-3), 72.6 (C-5), 74.8 (C-1'), 77.0 (C-4), 109.2 [$\underline{C}(CH_3)_2$], 127.8 (CH-Ph), 128.6 (CH-Ph), 129.3 (CH-Ph), 136.9 (C-Ph); HRMS (ESI): calcd. for $C_{15}H_{22}NO_4$ $[M+H]^+$ 280.1544; found 280.1541.

(±)-2,2,2-Trichloroethyl 4-(benzoyloxy)-3-isopropylisoxazolidine-2-carboxylate (11)

2,2,2-Trichloroethyl chloroformate (0.33 mL, 2.4 mmol) was added dropwise to a stirred solution of *N*-benzylisoxazolidine **7b** (260 mg, 0.8 mmol) and lithium iodide (160 mg, 1.2 mmol) in anhydrous acetonitrile (4 mL) under argon. The reaction mixture was stirred at 60 °C for 8 h. When TLC showed that the starting isoxazolidine disappeared (TLC; hexanes/EtOAc, 9:1), a sat. aq $NaHCO_3$ solution (10 mL) and CH_2Cl_2 (20 mL) were added. After vigorous stirring for additional 5 min, the organic layer was separated, and the aqueous phase was extracted with CH_2Cl_2 (10 mL). The combined organic layers were washed with water (20 mL), dried over $MgSO_4$ and concentrated under reduced pressure. The product was purified by FCC (hexanes/EtOAc, 9:1) to give *N*-Troc-isoxazolidine **11** (230 mg, 0.56 mmol, 70%) as a colourless sticky oil. R_f = 0.20 (*n*-hexane/EtOAc, 9:1); IR (ATR): υ_{max} = 3067, 2963, 2881, 1755, 1717, 1374, 1315, 1265, 1107, 1052, 805, 708, 572 cm^{-1}; ^{1}H NMR (600 MHz, $CDCl_3$) δ 1.09 (d, *J* = 6.7 Hz, 3H, CH_3), 1.14 (d, 3H, *J* = 6.7 Hz, CH_3), 1.89–1.97 [m, 1H, $\underline{CH}(CH_3)_2$], 4.12 (dd, *J* = 3.4, 9.5 Hz, 1H, H-5a), 4.22 (d, *J* = 8.4 Hz, 1H, H-3), 4.51 (dd, *J* = 5.9, 9.6 Hz, 1H, H-5b), 4.72 (d, *J* = 11.9 Hz, 1H, Cl_3CCH_2O), 4.86 (d, *J* = 11.9 Hz, 1H, Cl_3CCH_2O), 5.64 (ddd, *J* = 1.1, 3.5, 5.9 Hz, 1H, H-4), 7.43–7.46 (m, 2H, H-Ph), 7.57–7.60 (m, 1H, H-Ph), 7.95–7.98 (m, 2H, H-Ph); ^{13}C NMR (150 MHz, $CDCl_3$) δ 19.1 (CH_3), 19.2 (CH_3), 29.7 [$\underline{C}H(CH_3)_2$], 72.1 (C-3), 74.4, 75.3 (C-5, $CO_2\underline{C}H_2$), 78.9 (C-4), 94.8 (CCl_3), 128.6 (CH-Ph), 128.9 (C-Ph), 129.7 (CH-Ph), 133.7 (CH-Ph), 157.1 ($\underline{C}O_2CH_2$), 165.9 ($\underline{C}$OPh); HRMS (ESI): calcd. for $C_{16}H_{19}Cl_3NO_5$ $[M+H]^+$ 410.0324; found 410.0329.

(±)-3-Isopropylisoxazolidin-4-ol (12)

The NaOH solution (0.6 mL, 3.6 mmol, 6 M) was added to a solution of *N*-Troc-isoxazolidine **11** (120 mg, 0.29 mmol) in isopropyl alcohol (1.2 mL) and the mixture was stirred at rt for 1 h. After the disappearance of the starting material (TLC, hexanes/EtOAc, 5:1), the solution was neutralized with HCl (6 M). Afterwards, a sat. aq $NaHCO_3$ solution (2 mL) and solid NaCl were added and the resulting slurry was vigorously extracted with EtOAc (3 × 5 mL). The combined organic layers were dried over $MgSO_4$ and concentrated under reduced pressure. The product was purified by FCC (EtOAc) to give isoxazolidinol **12** (28 mg, 0.21 mmol, 72%) as a colourless sticky oil. R_f = 0.25 (EtOAc); IR (ATR): υ_{max} = 3160, 2971, 2899, 2874, 1473, 1093, 1036, 1013, 935, 886, 754, 718, 647 cm^{-1}; ^{1}H NMR (600 MHz, $CDCl_3$) δ 1.02 (d, *J* = 6.7 Hz, 3H, CH_3), 1.04 (d, *J* = 6.7 Hz, 3H, CH_3), 1.58–1.66 [m, 1H, $\underline{CH}(CH_3)_2$], 2.90 (dd, *J* = 1.8, 8.8 Hz, 1H, H-3), 3.83 (dd, *J* = 1.9, 9.4 Hz, 1H, H-5a), 3.89 (dd, *J* = 5.0, 9.4 Hz, 1H, H-5b), 4.51 (dt, *J* = 2.0, 5.0 Hz, 1H, H-4); ^{13}C NMR (150 MHz,

$CDCl_3$) δ 19.8 (CH_3), 20.0 (CH_3), 29.1 [<u>C</u>H(CH_3)$_2$], 75.7 (C-3), 78.0 (C-5), 78.4 (C-4); HRMS (ESI): calcd. for $C_6H_{14}NO_2$ $[M+H]^+$ 132.1020; found 132.1020.

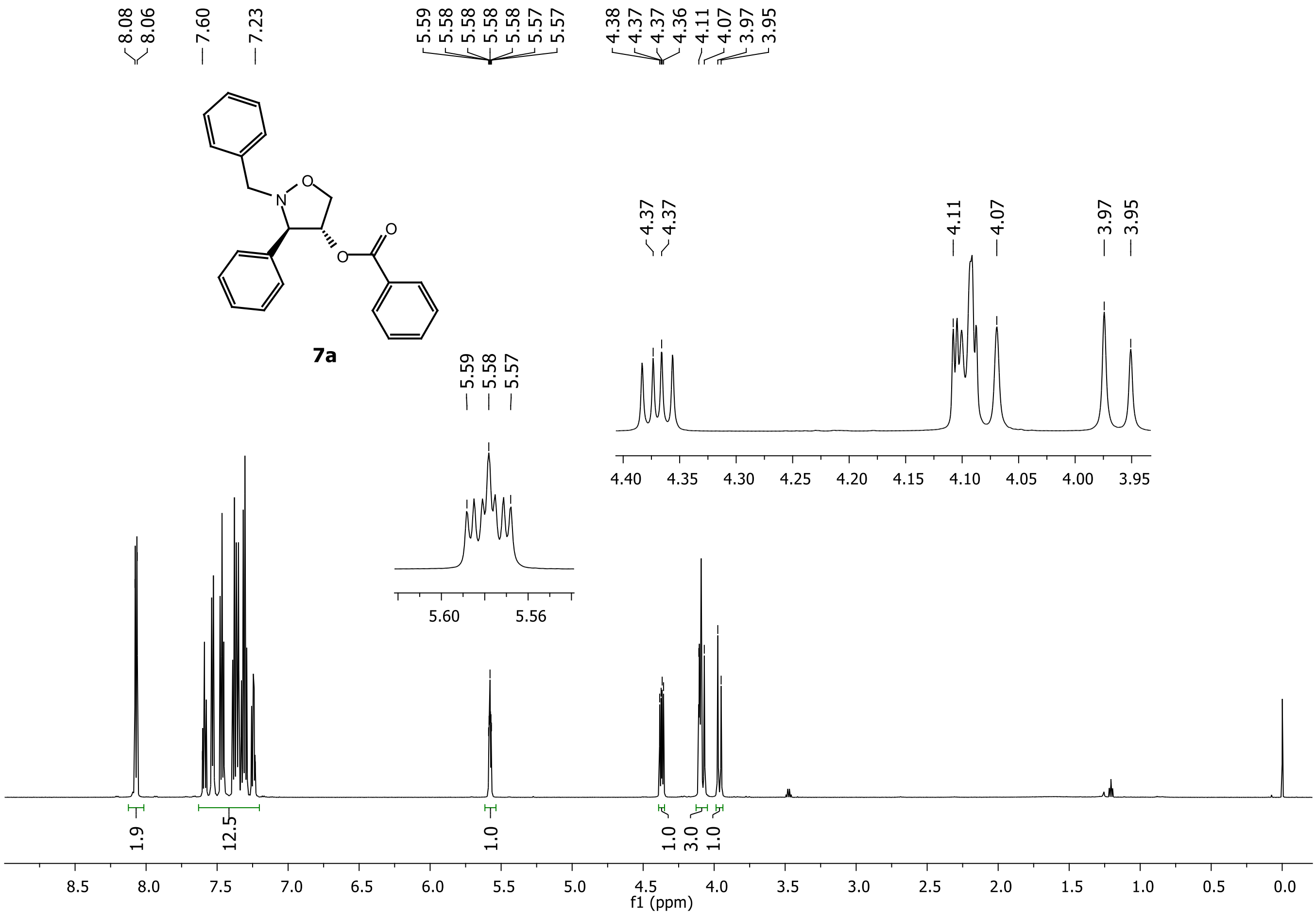
8.08
8.06
7.60
7.23
5.59
5.58
5.58
5.58
5.58
5.57
5.57
4.38
4.37
4.37
4.36
4.11
4.07
3.97
3.95
7a
1.9
12.5
1.0
1.0
3.0
1.0
f1 (ppm)

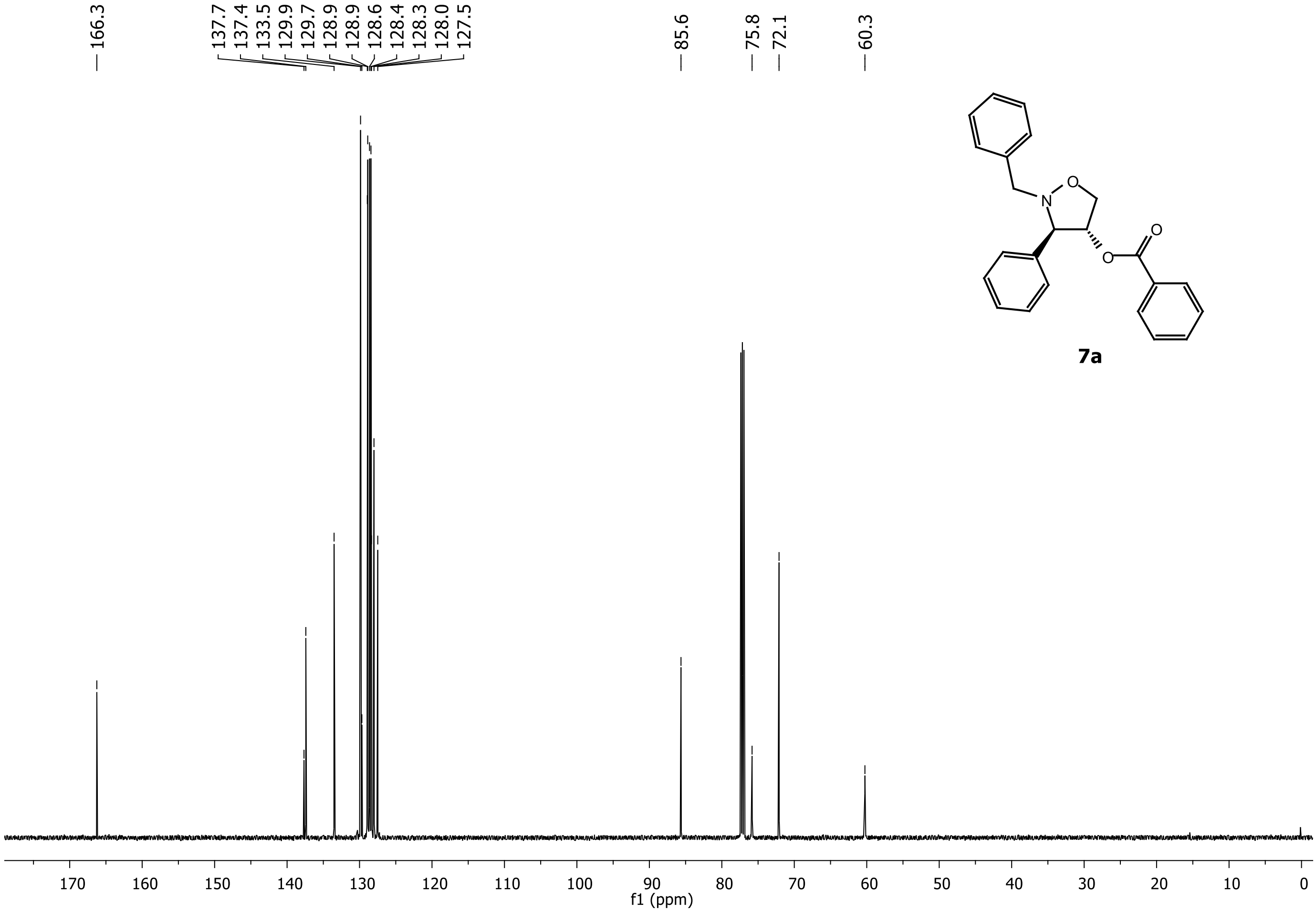
166.3
137.7
137.4
133.5
129.9
129.7
128.9
128.9
128.6
128.4
128.3
128.0
127.5
85.6
75.8
72.1
60.3
7a
f1 (ppm)

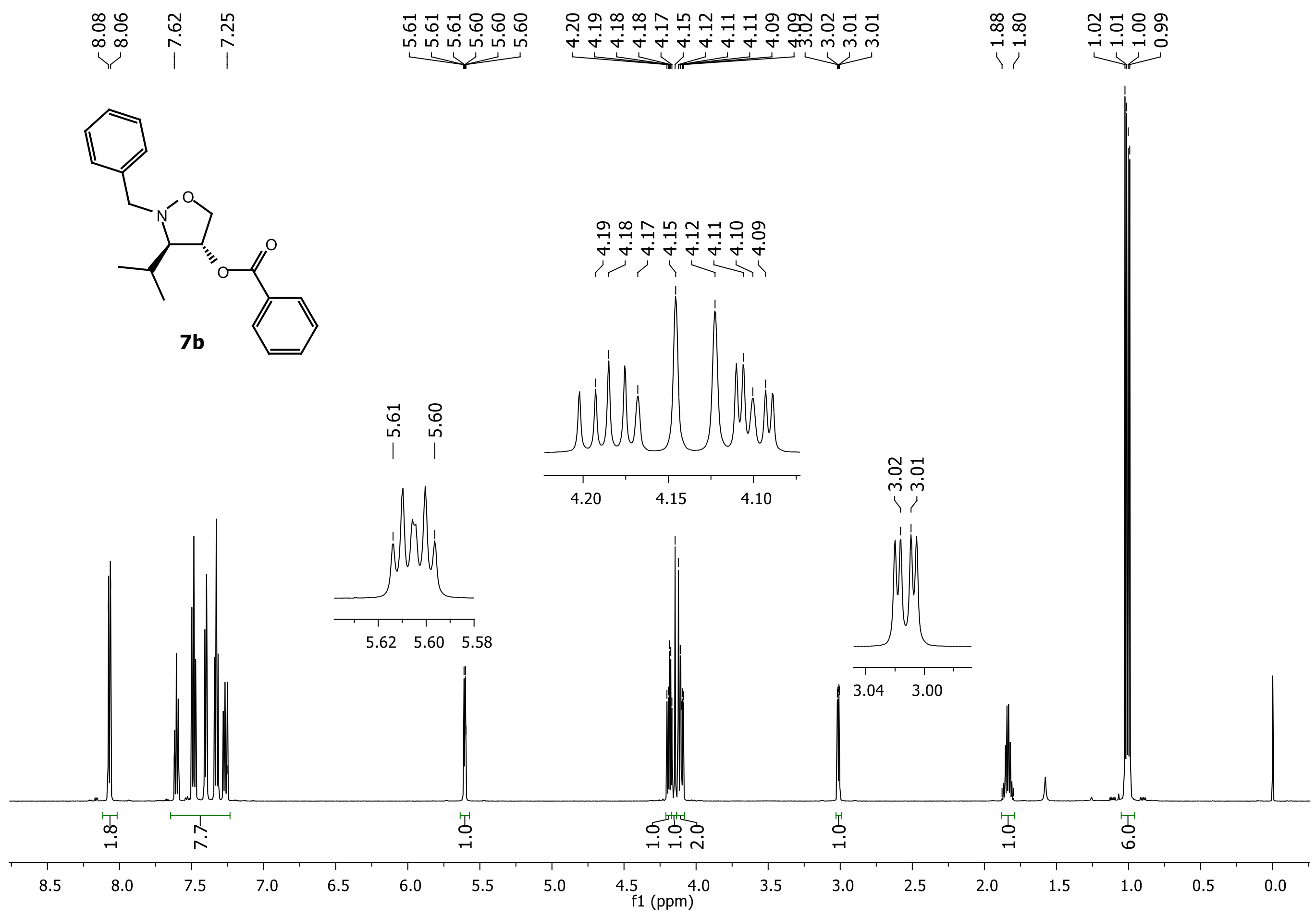
8.08
8.06
7.62
7.25
5.61
5.61
5.61
5.60
5.60
5.60
4.20
4.19
4.18
4.18
4.17
4.15
4.12
4.11
4.11
4.11
4.09
4.09
3.02
3.02
3.01
3.01
1.88
1.80
1.02
1.01
1.00
0.99
7b
1.8
7.7
1.0
1.0
1.0
2.0
1.0
1.0
6.0
f1 (ppm)

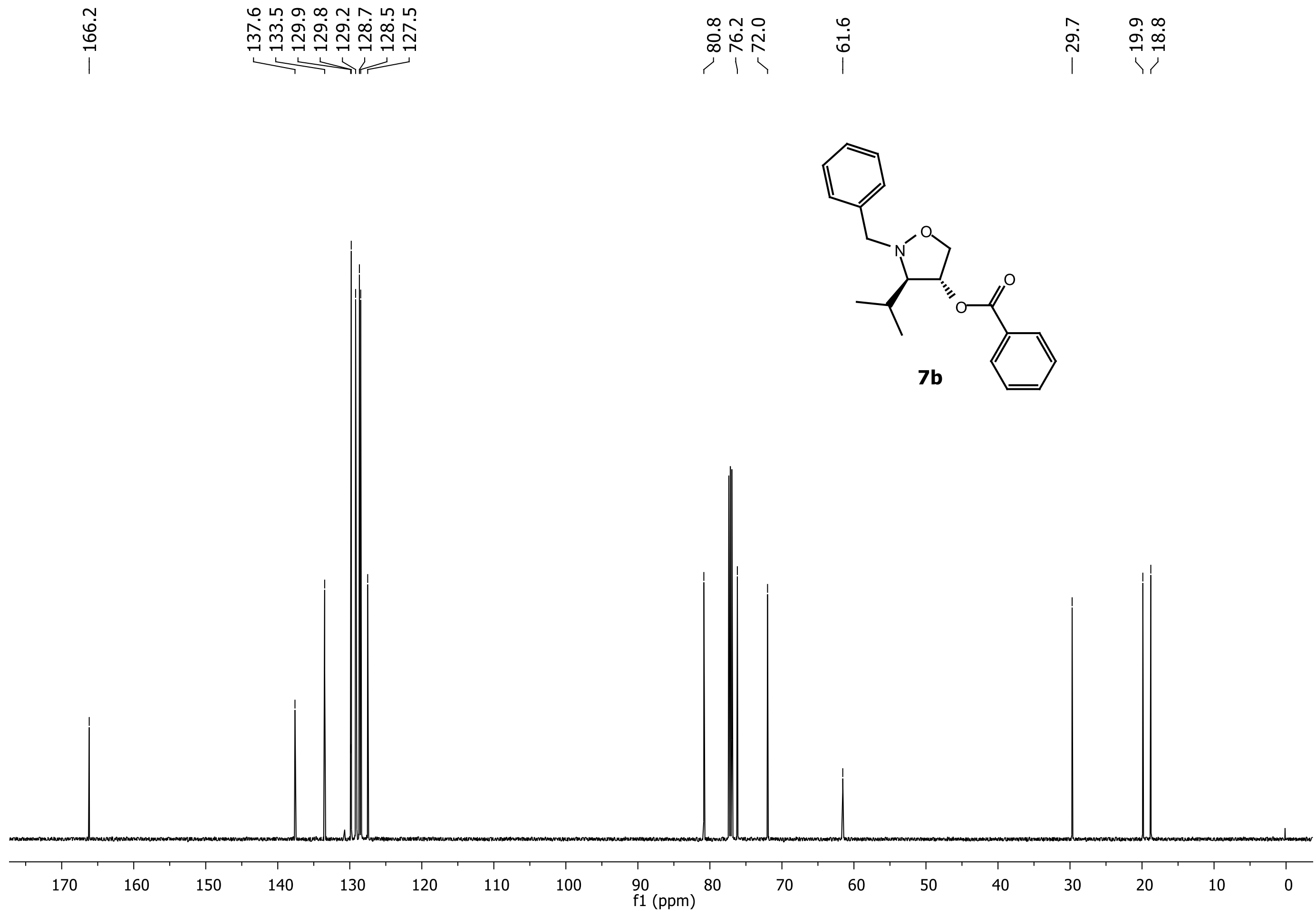
166.2
137.6
133.5
129.9
129.8
129.2
128.7
128.5
127.5
80.8
76.2
72.0
61.6
29.7
19.9
18.8
7b
f1 (ppm)

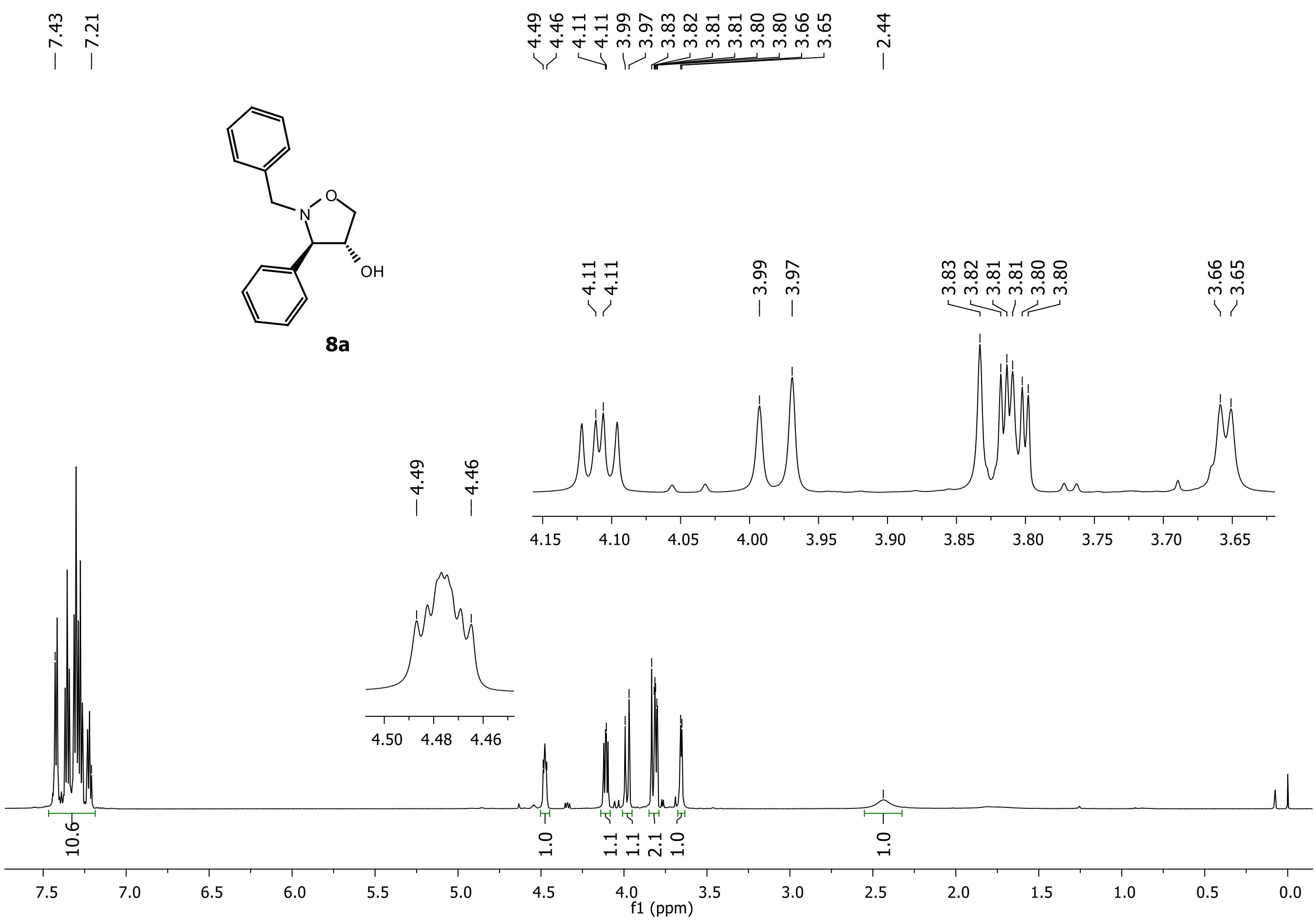
7.43
7.21
4.49
4.46
4.11
4.11
3.99
3.97
3.83
3.82
3.81
3.81
3.80
3.80
3.66
3.65
2.44
N
O
OH
8a
10.6
1.0
1.1
1.1
2.1
1.0
1.0
f1 (ppm)

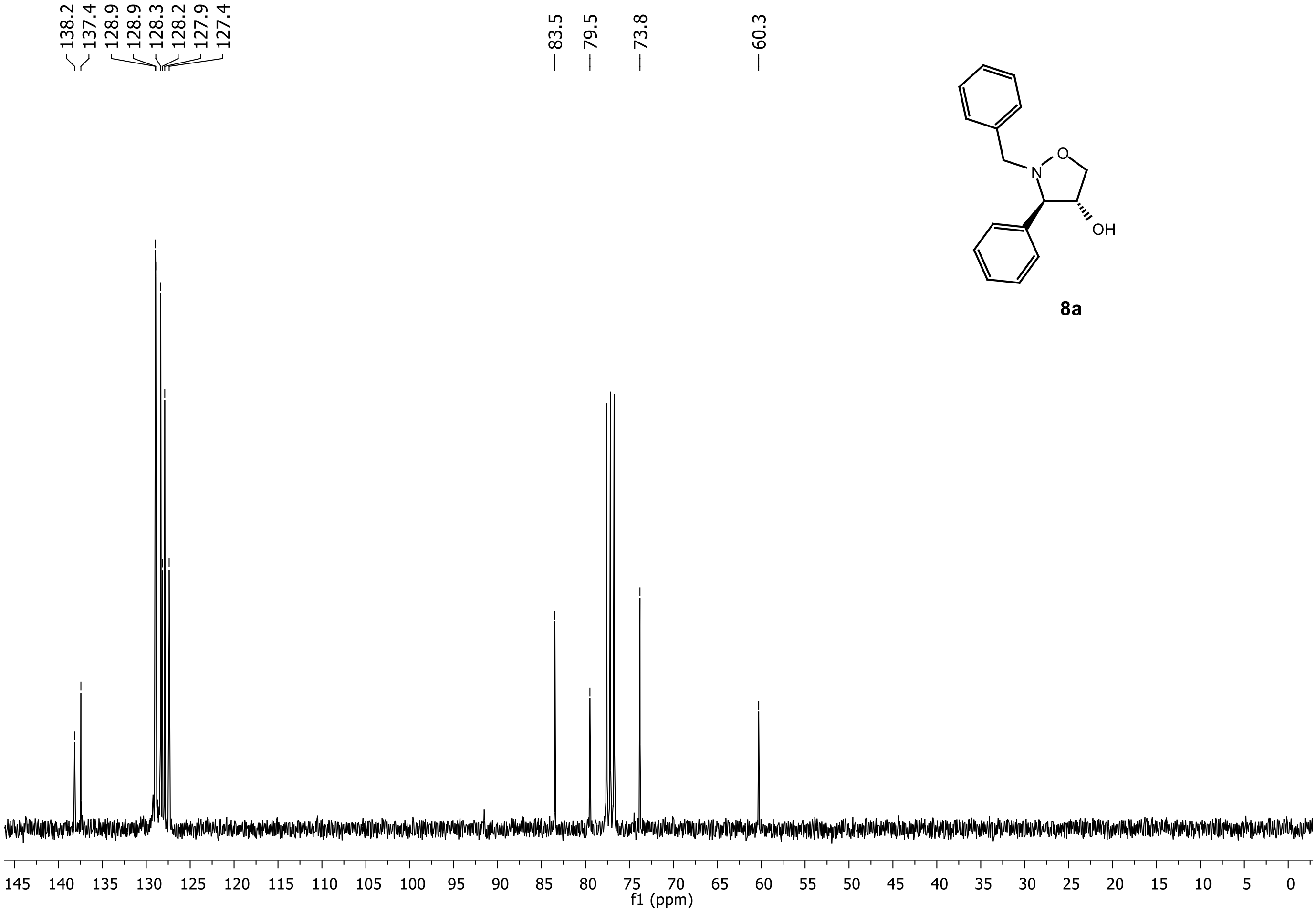
138.2
137.4
128.9
128.9
128.3
128.2
127.9
127.4
83.5
79.5
73.8
60.3
N
O
OH
8a
f1 (ppm)

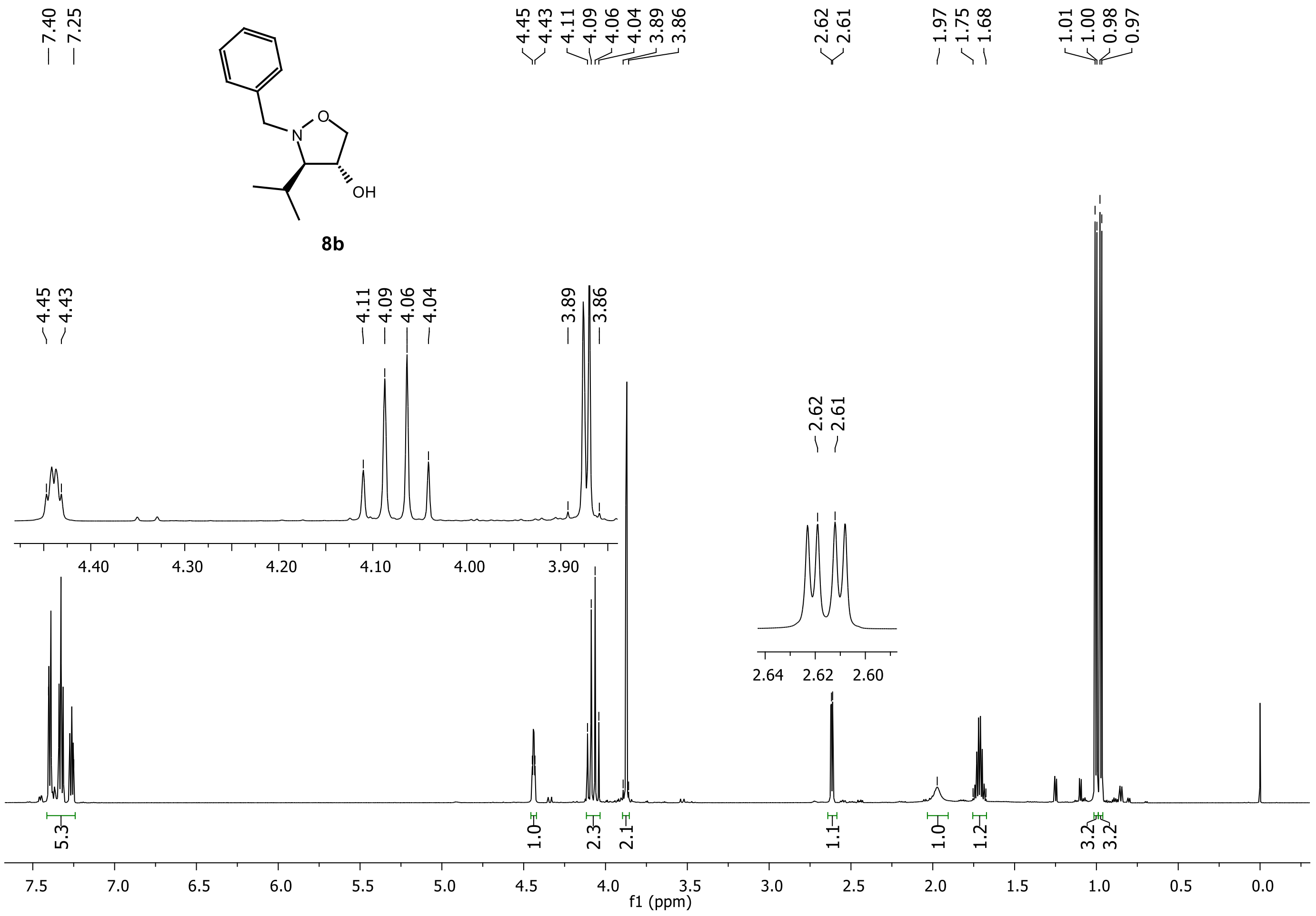
7.40
7.25
4.45
4.43
4.11
4.09
4.06
4.04
3.89
3.86
2.62
2.61
1.97
1.75
1.68
1.01
1.00
0.98
0.97
N
O
OH
8b
4.40
4.30
4.20
4.10
4.00
3.90
2.64
2.62
2.60
5.3
1.0
2.3
2.1
1.1
1.0
1.2
3.2
3.2
7.5 7.0 6.5 6.0 5.5 5.0 4.5 4.0 3.5 3.0 2.5 2.0 1.5 1.0 0.5 0.0
f1 (ppm)

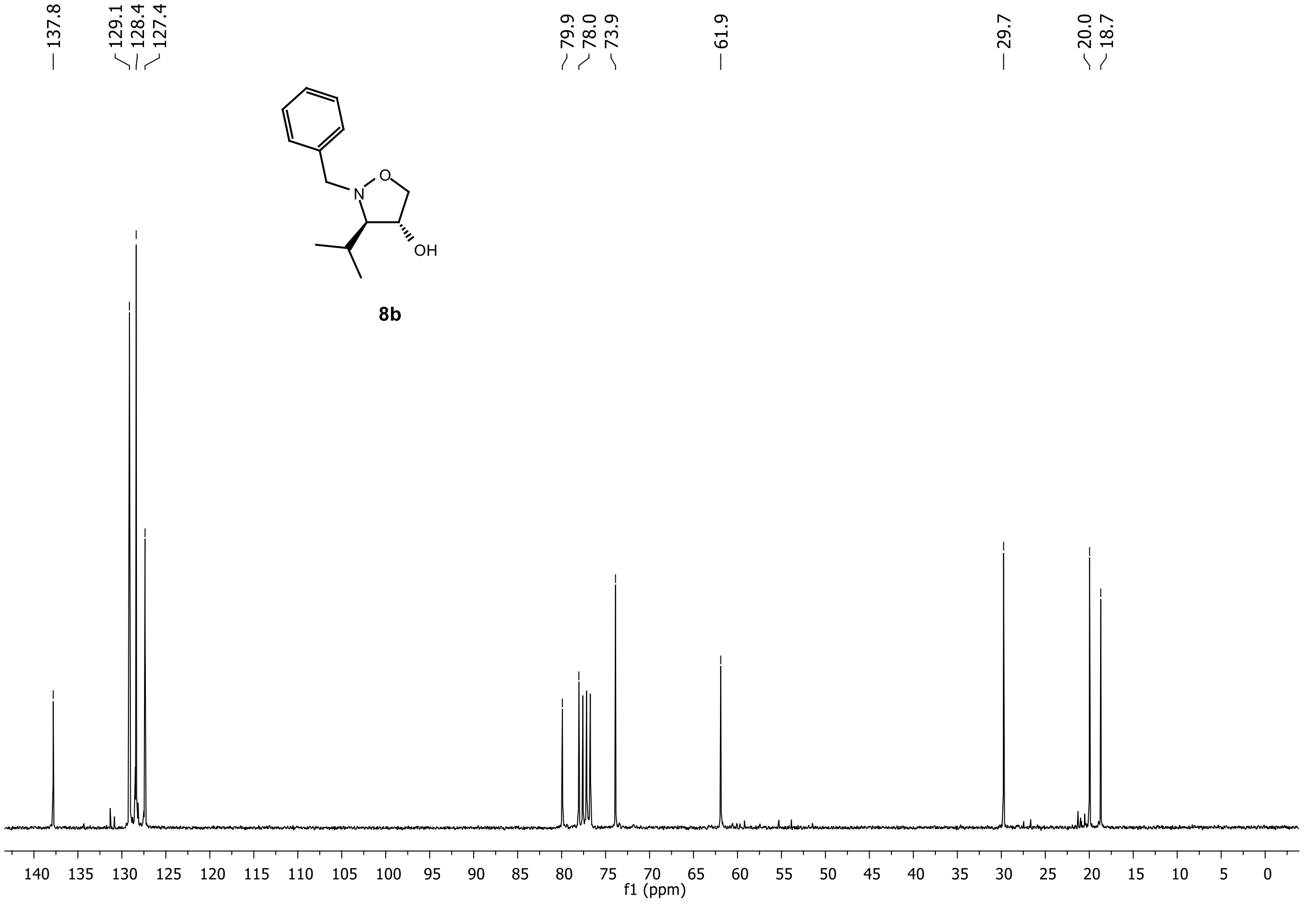
137.8
129.1
128.4
127.4
79.9
78.0
73.9
61.9
29.7
20.0
18.7
N
O
OH
8b
140 135 130 125 120 115 110 105 100 95 90 85 80 75 70 65 60 55 50 45 40 35 30 25 20 15 10 5 0
f1 (ppm)

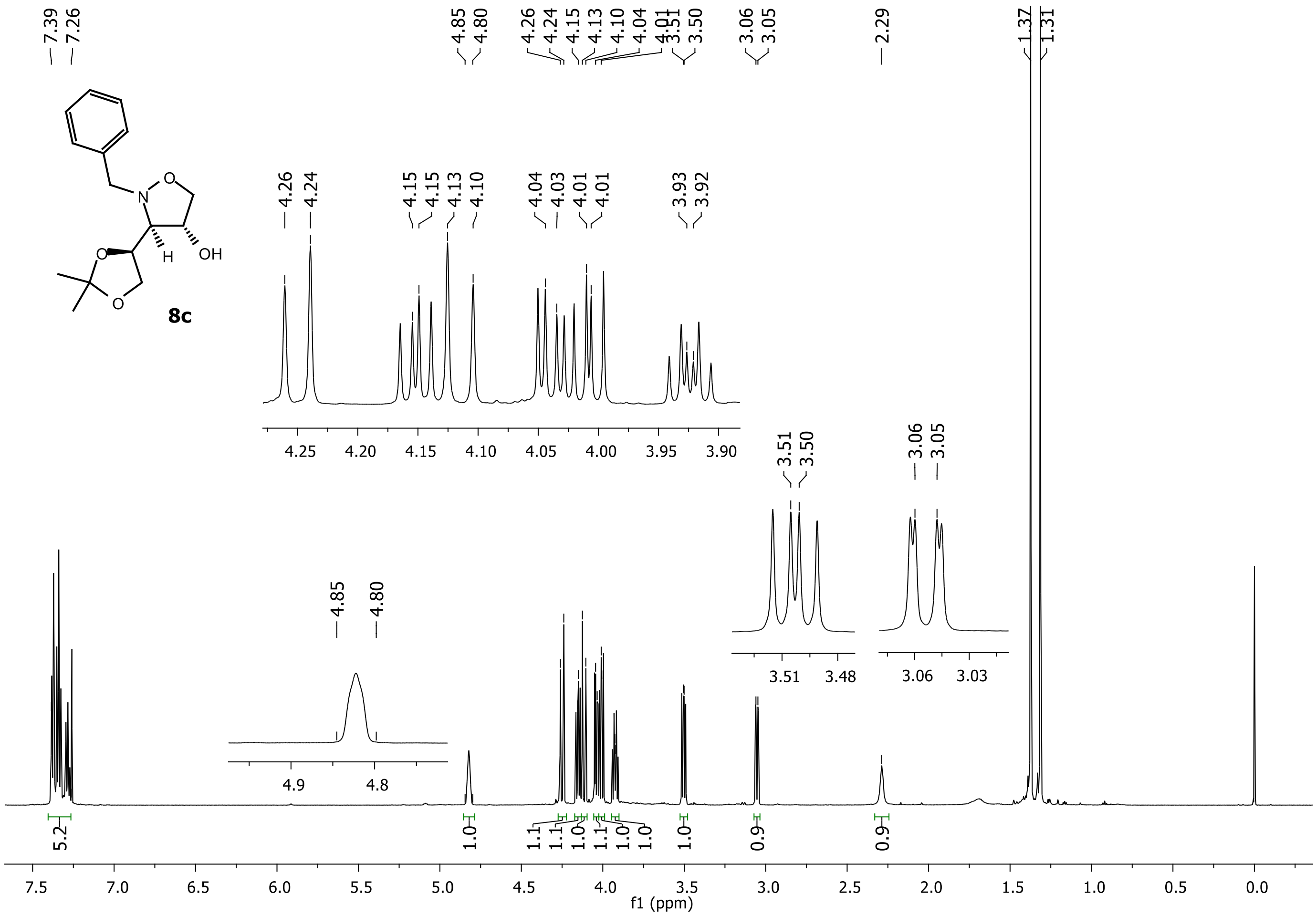
7.39
7.26
4.85
4.80
4.26
4.24
4.15
4.13
4.10
4.04
4.01
3.51
3.50
3.06
3.05
2.29
1.37
1.31
OH
H
N
O
8c
5.2
1.0
1.1
1.1
1.0
1.1
1.0
1.0
1.0
0.9
0.9
f1 (ppm)

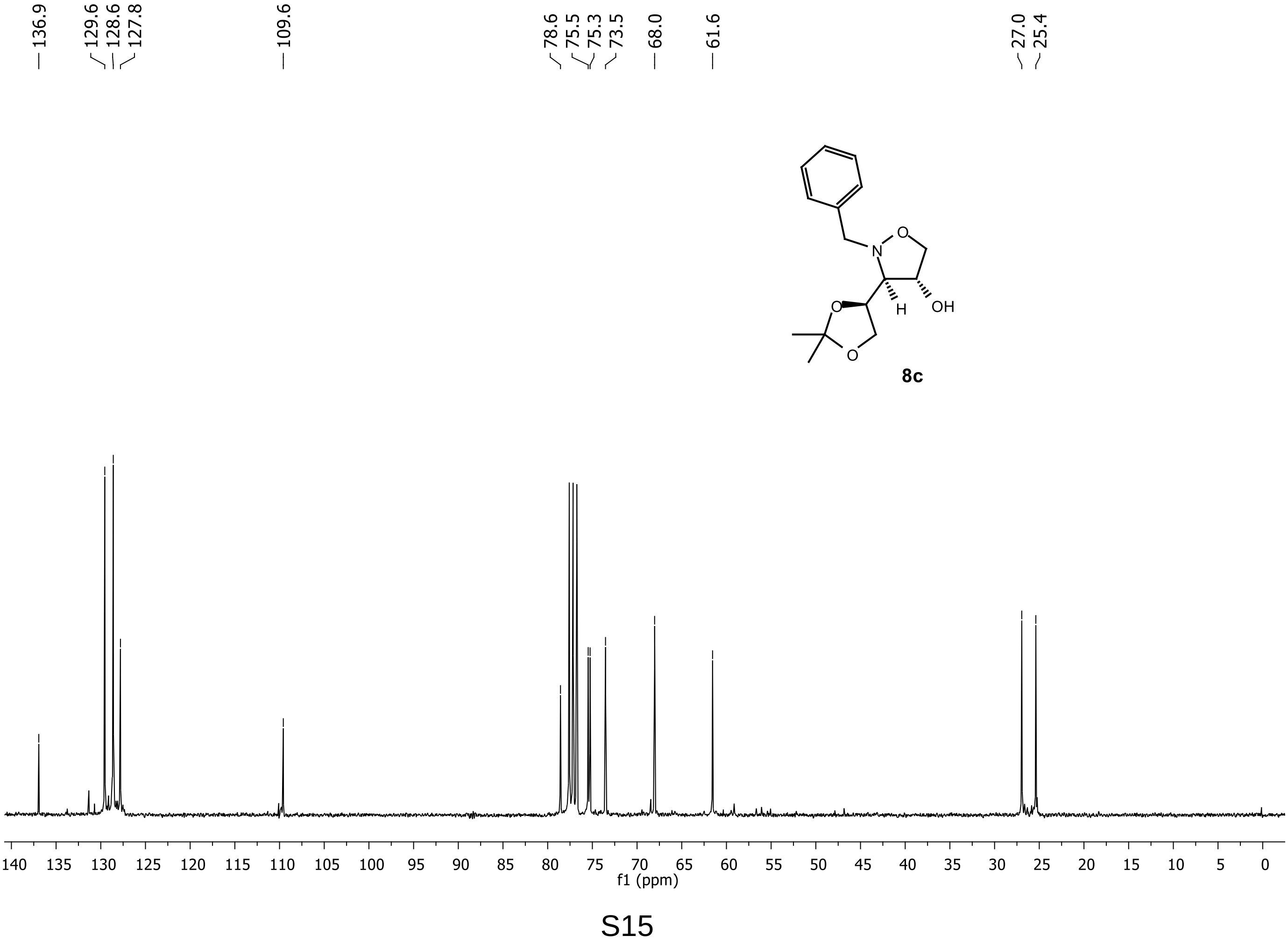
136.9
129.6
128.6
127.8
109.6
78.6
75.5
75.3
73.5
68.0
61.6
27.0
25.4
8c
f1 (ppm)

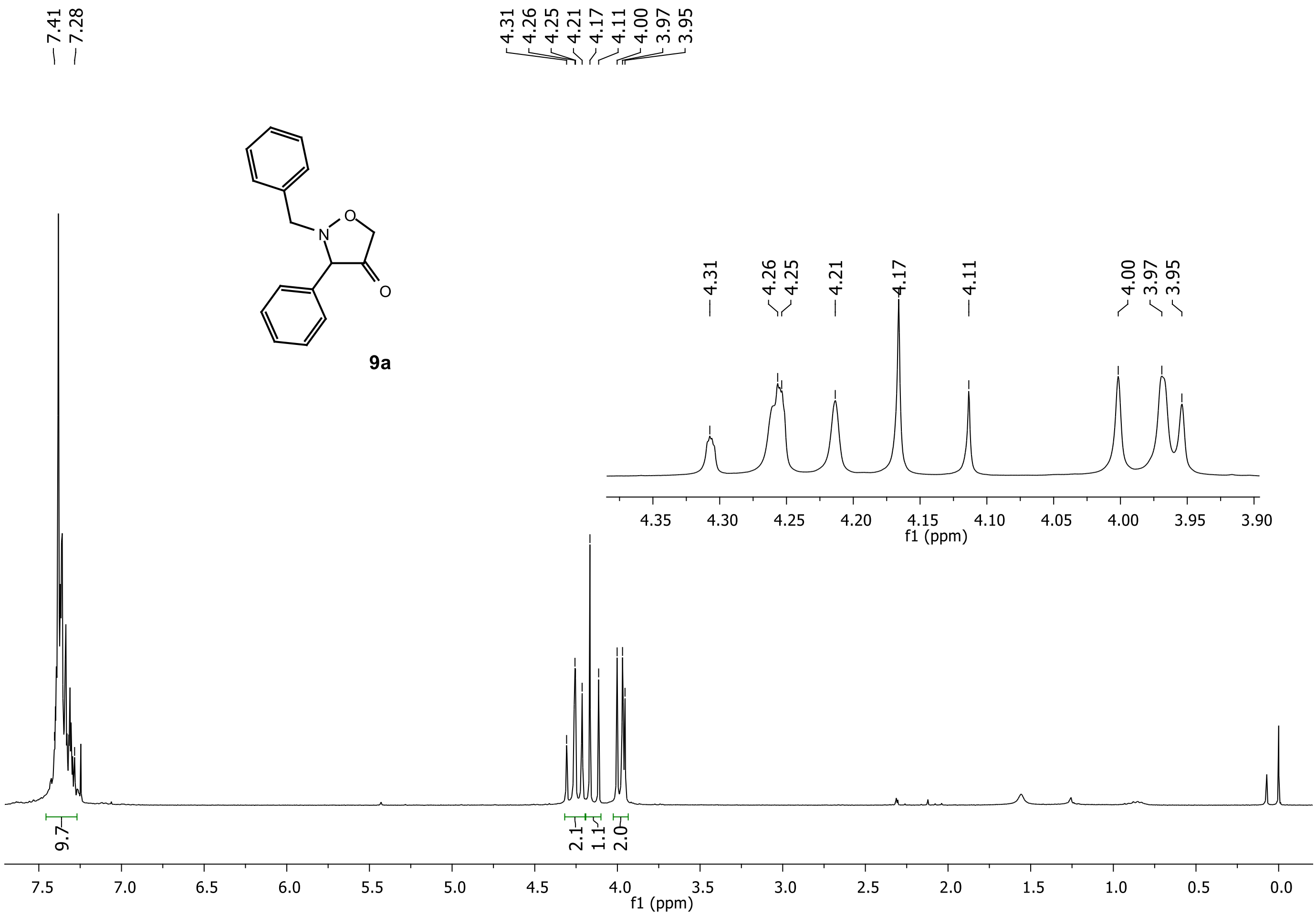
7.41
7.28
4.31
4.26
4.25
4.21
4.17
4.11
4.00
3.97
3.95
O
N
O
9a
9.7
2.1
1.1
2.0
f1 (ppm)

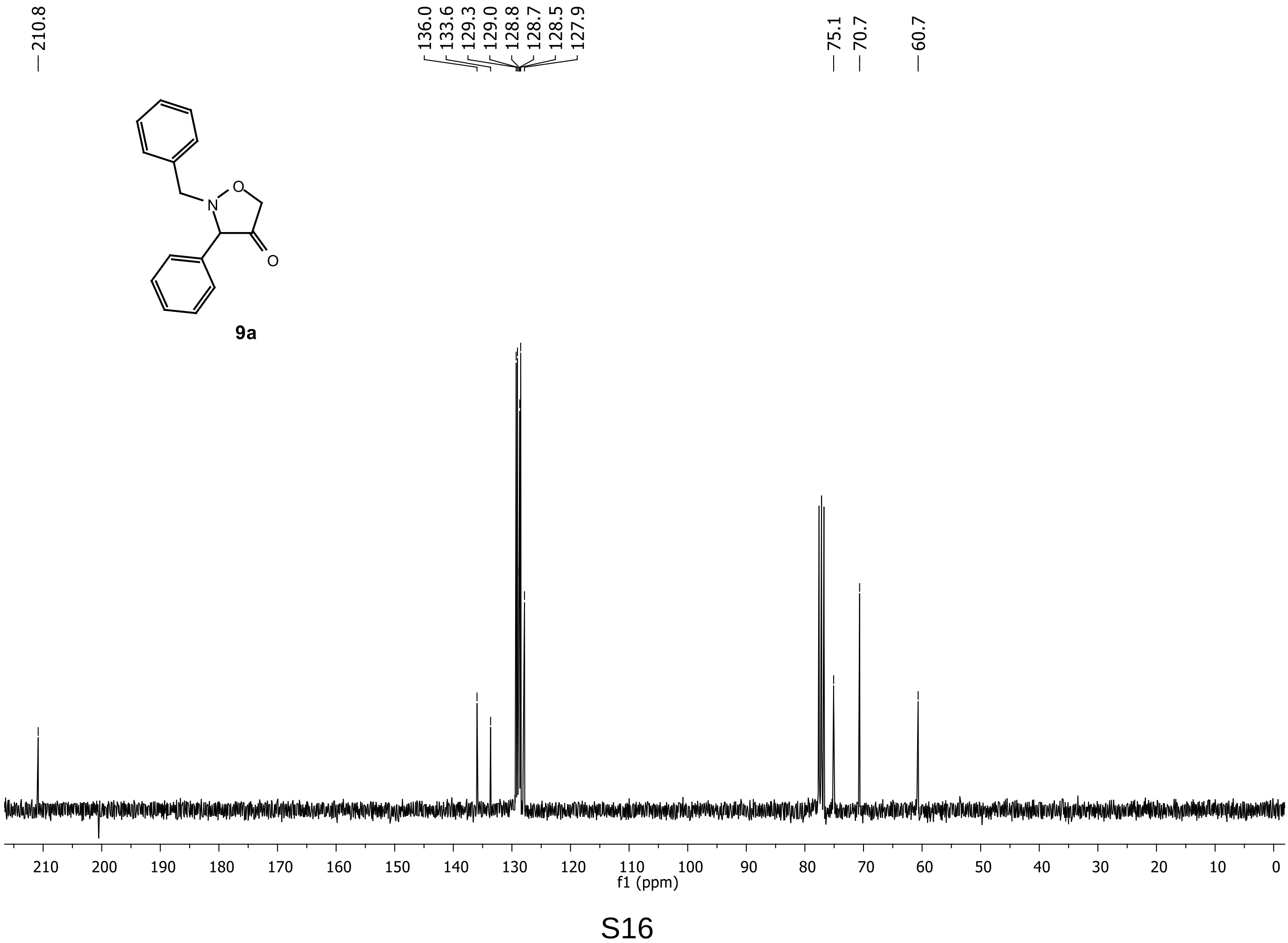
210.8
136.0
133.6
129.3
129.0
128.8
128.7
128.5
127.9
75.1
70.7
60.7
O
N
O
9a
f1 (ppm)

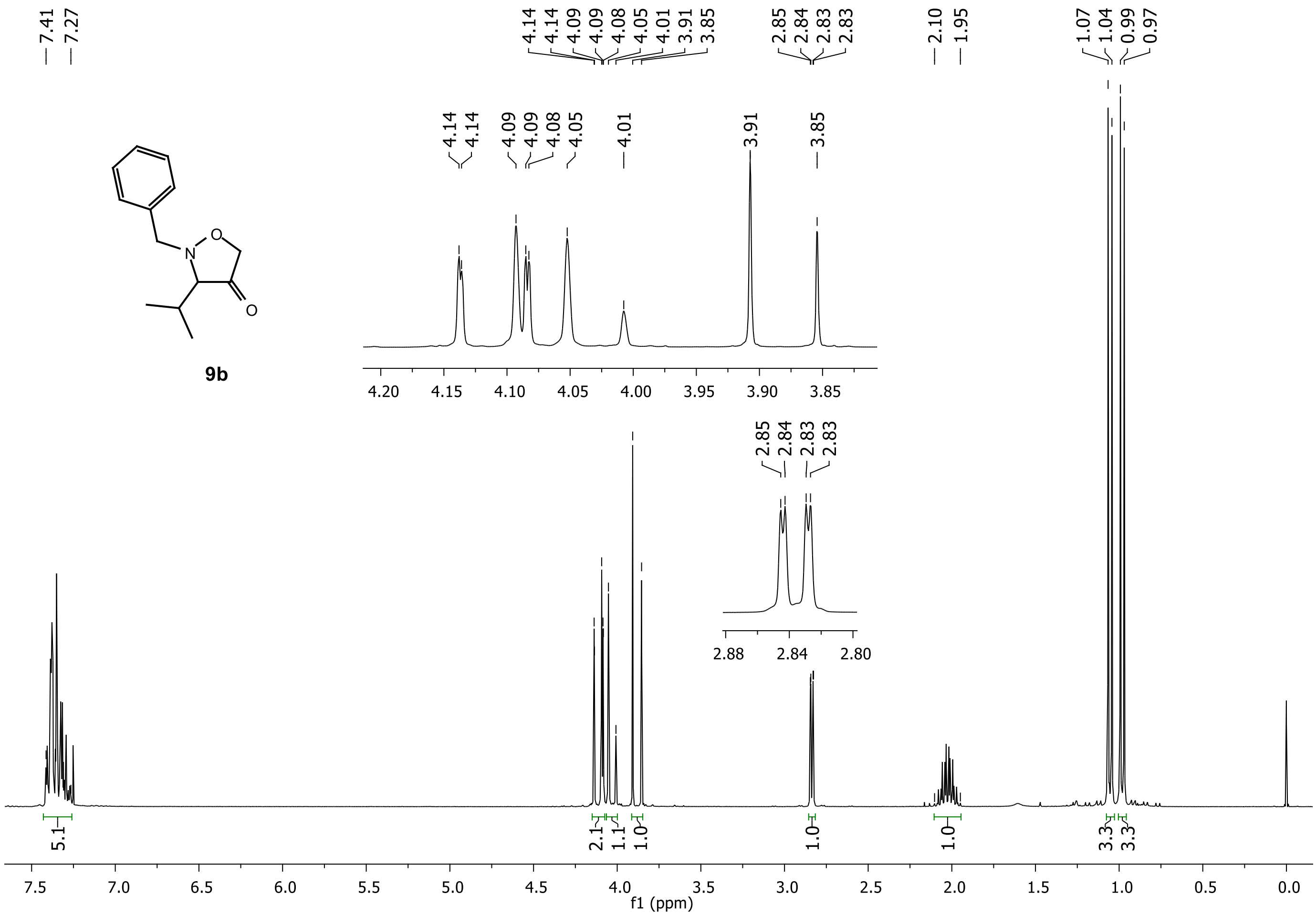
7.41
7.27
4.14
4.14
4.09
4.09
4.08
4.05
4.01
3.91
3.85
2.85
2.84
2.83
2.83
2.10
1.95
1.07
1.04
0.99
0.97
9b
5.1
2.1
1.1
1.0
1.0
1.0
3.3
3.3
f1 (ppm)

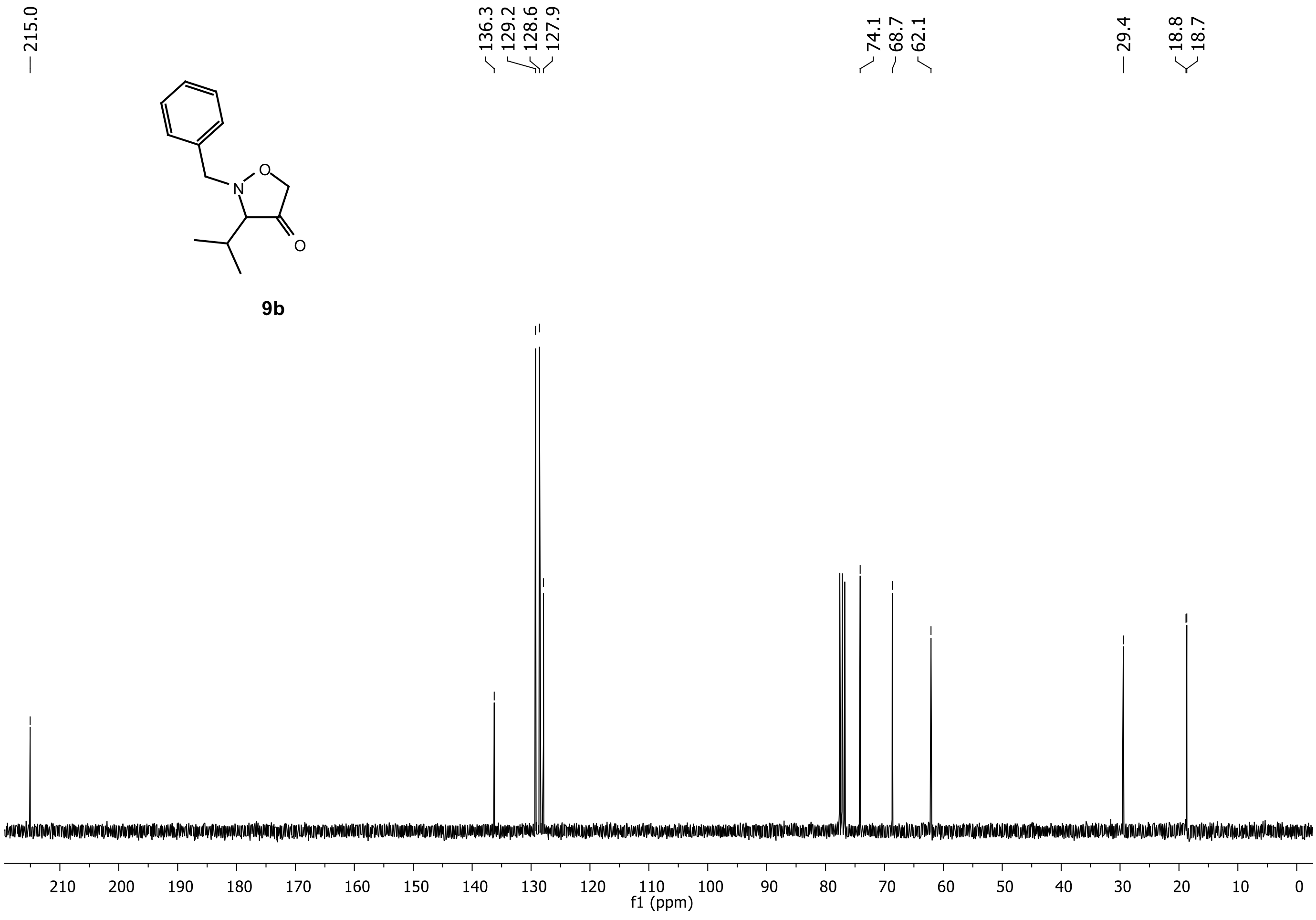
215.0
136.3
129.2
128.6
127.9
74.1
68.7
62.1
29.4
18.8
18.7
9b
f1 (ppm)

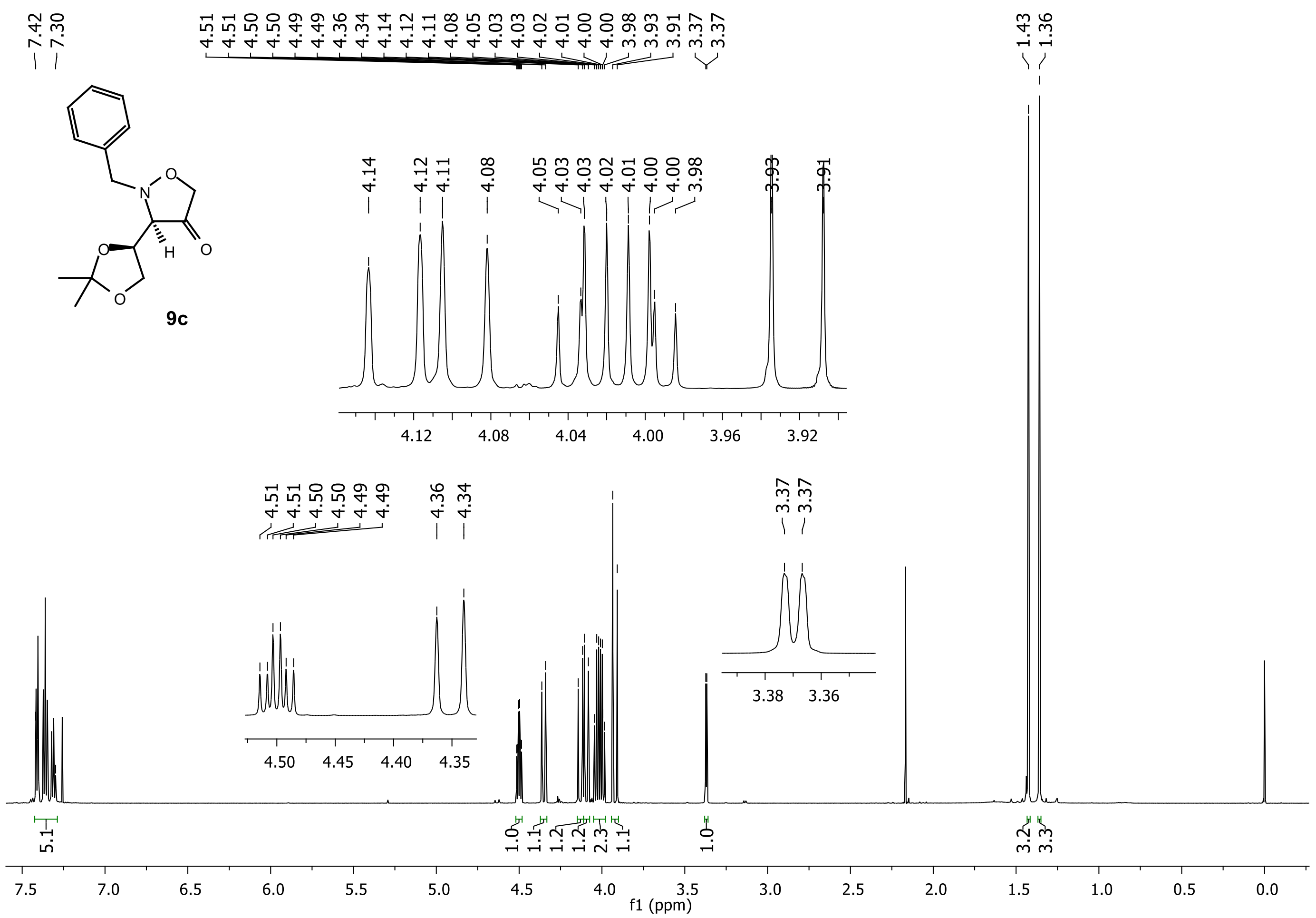

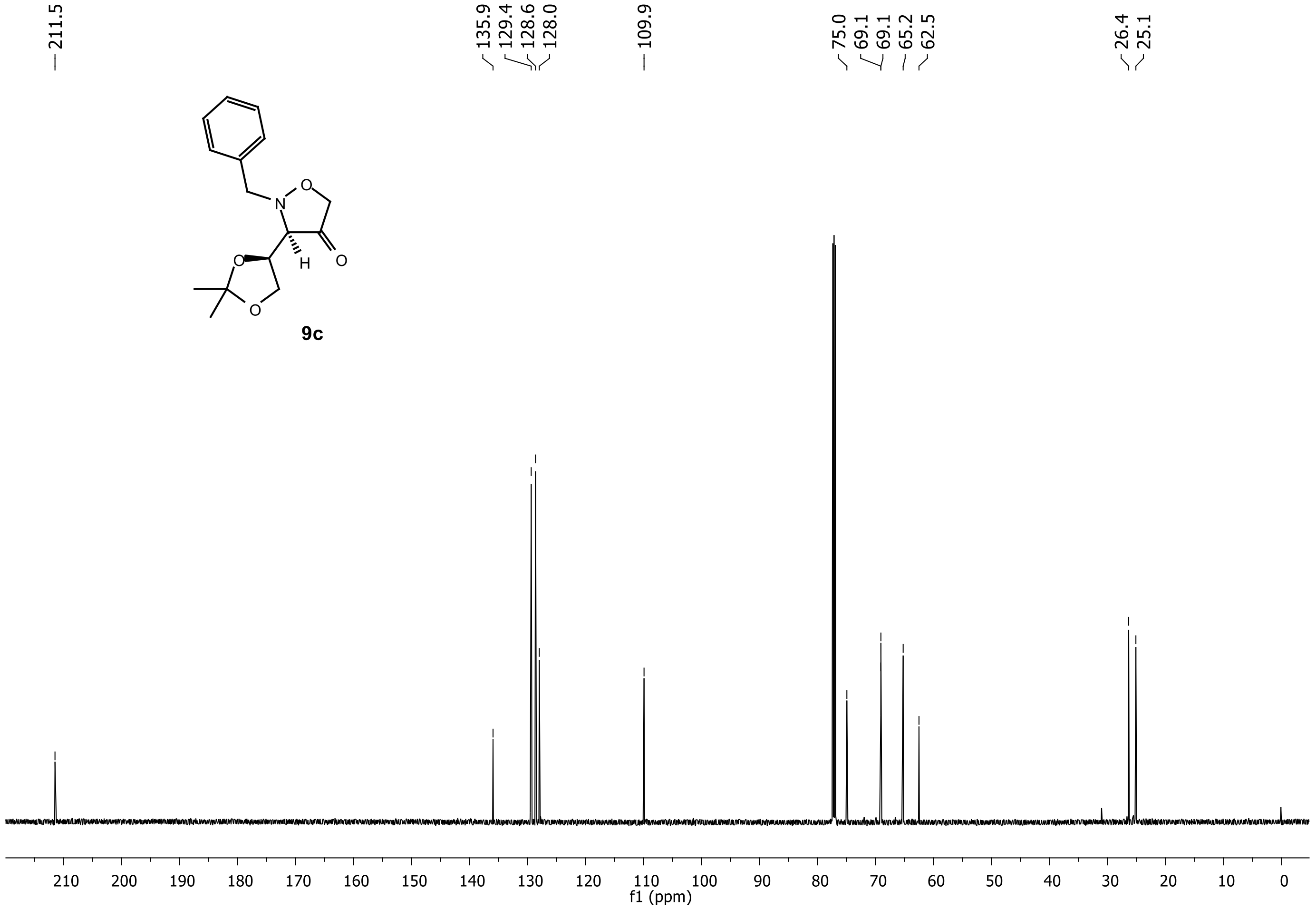

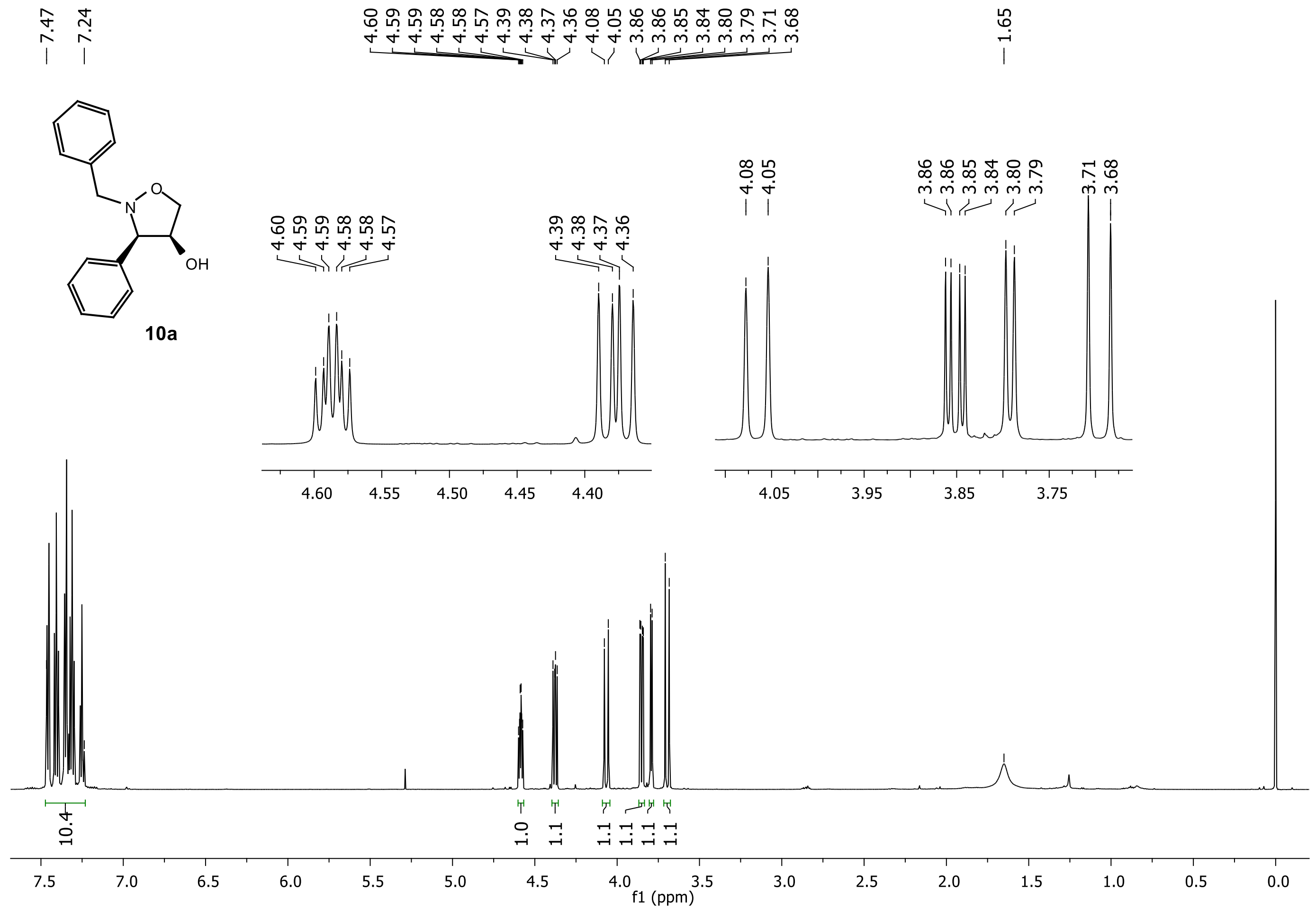
7.47
7.24
4.60 4.59 4.59 4.58 4.58 4.57 4.39 4.38 4.37 4.36 4.08 4.05 3.86 3.86 3.85 3.84 3.80 3.79 3.71 3.68
1.65
O
N
OH
10a
10.4
1.0
1.1
1.1
1.1
1.1
1.1
7.5 7.0 6.5 6.0 5.5 5.0 4.5 4.0 3.5 3.0 2.5 2.0 1.5 1.0 0.5 0.0
f1 (ppm)

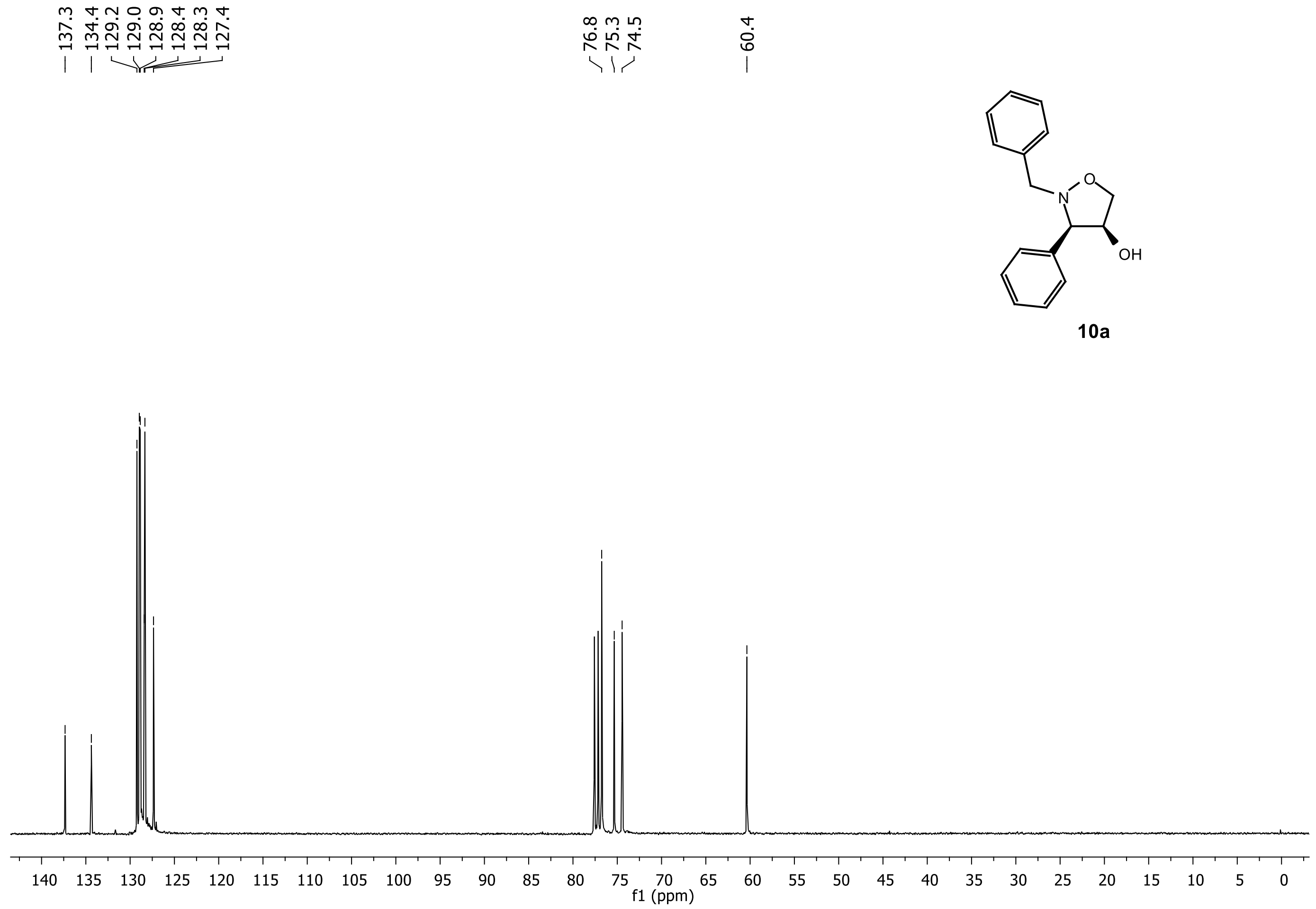
137.3
134.4
129.2 129.0 128.9 128.4 128.3 127.4
76.8 75.3 74.5
60.4
O
N
OH
10a
140 135 130 125 120 115 110 105 100 95 90 85 80 75 70 65 60 55 50 45 40 35 30 25 20 15 10 5 0
f1 (ppm)

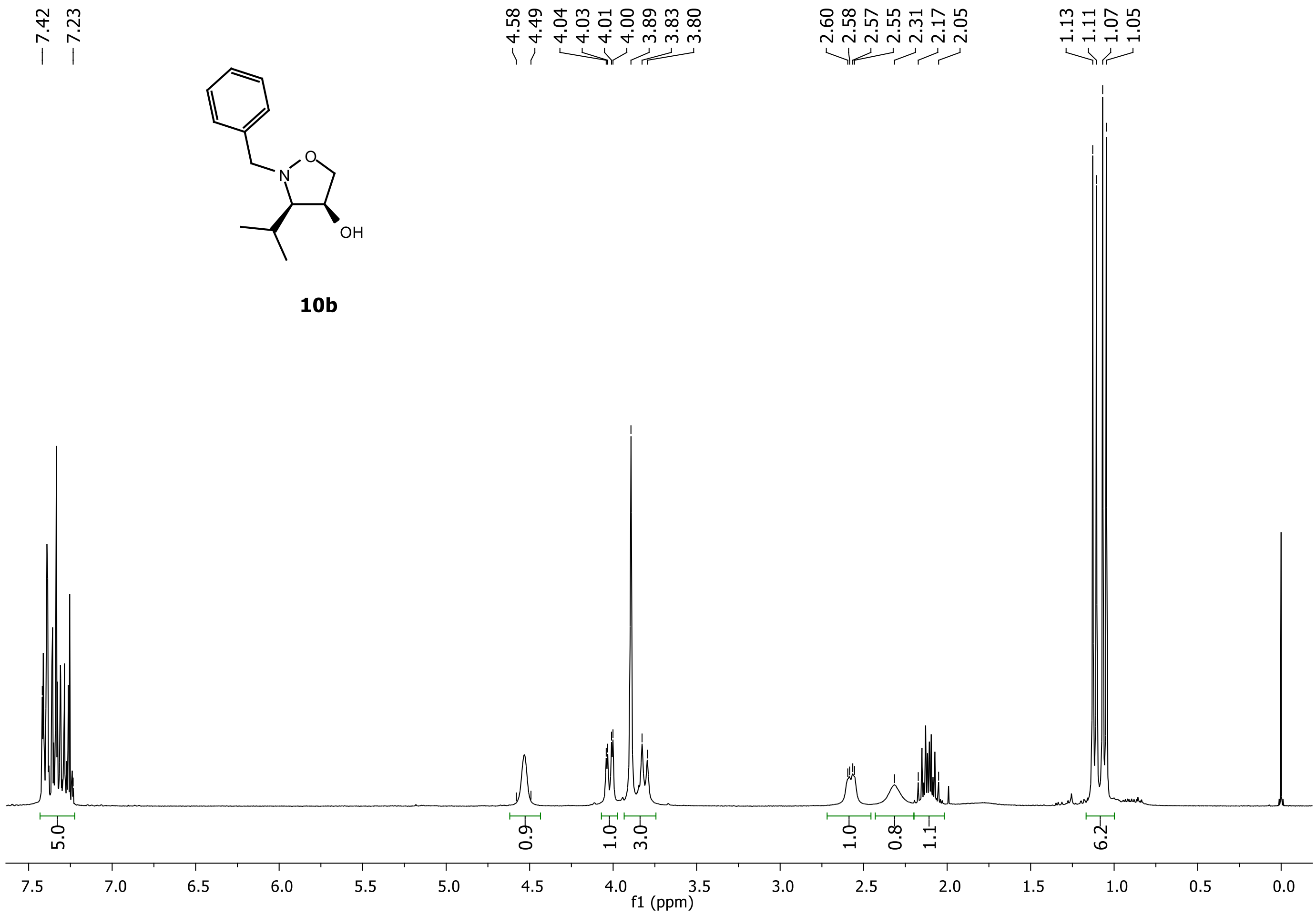

7.42
7.23
4.58
4.49
4.04
4.03
4.01
4.00
3.89
3.83
3.80
2.60
2.58
2.57
2.55
2.31
2.17
2.05
1.13
1.11
1.07
1.05
N
O
OH
10b
5.0
0.9
1.0
3.0
1.0
0.8
1.1
6.2
7.5 7.0 6.5 6.0 5.5 5.0 4.5 4.0 3.5 3.0 2.5 2.0 1.5 1.0 0.5 0.0
f1 (ppm)

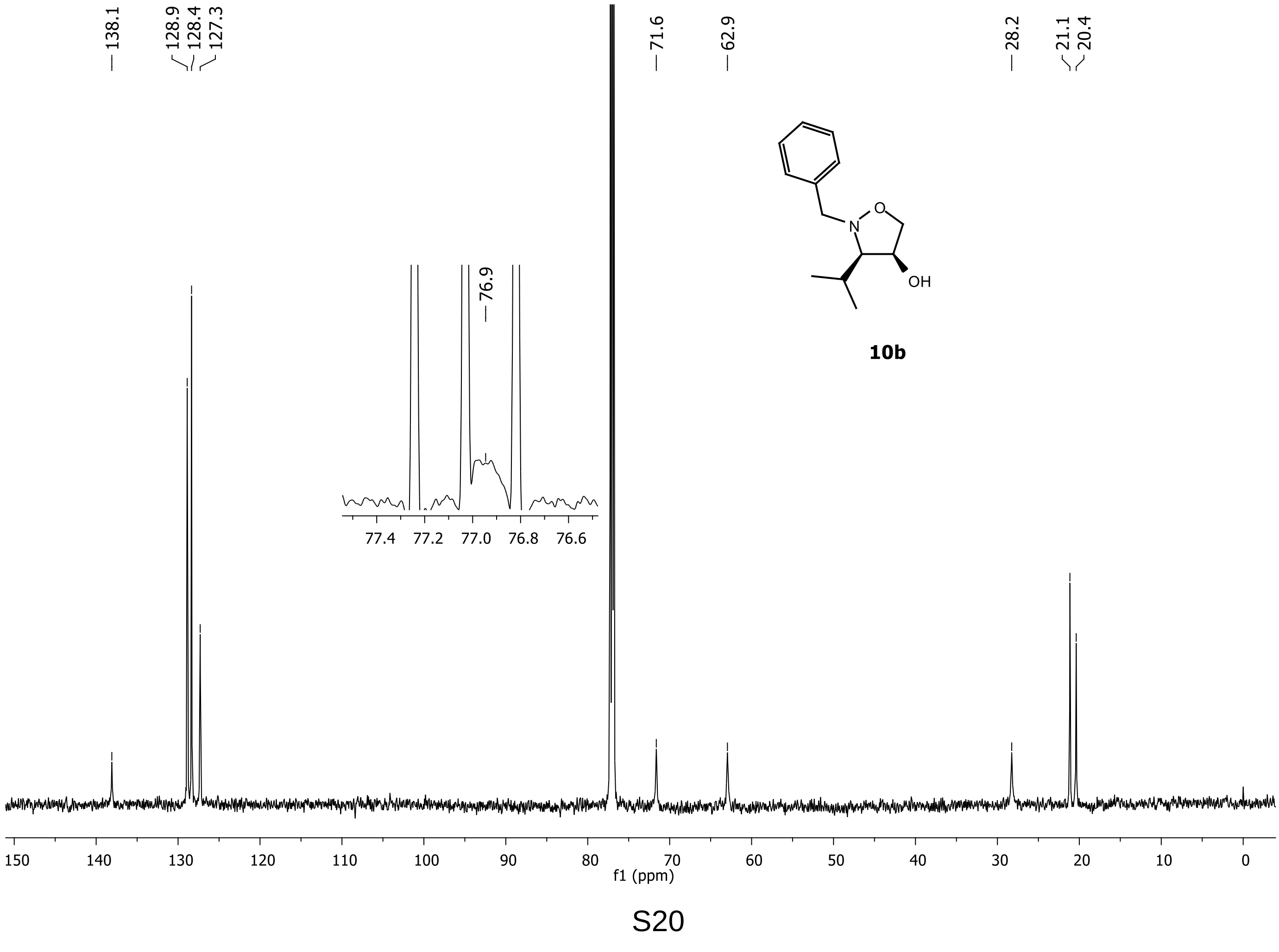

138.1
128.9
128.4
127.3
71.6
62.9
28.2
21.1
20.4
76.9
77.4 77.2 77.0 76.8 76.6
N
O
OH
10b
150 140 130 120 110 100 90 80 70 60 50 40 30 20 10 0
f1 (ppm)

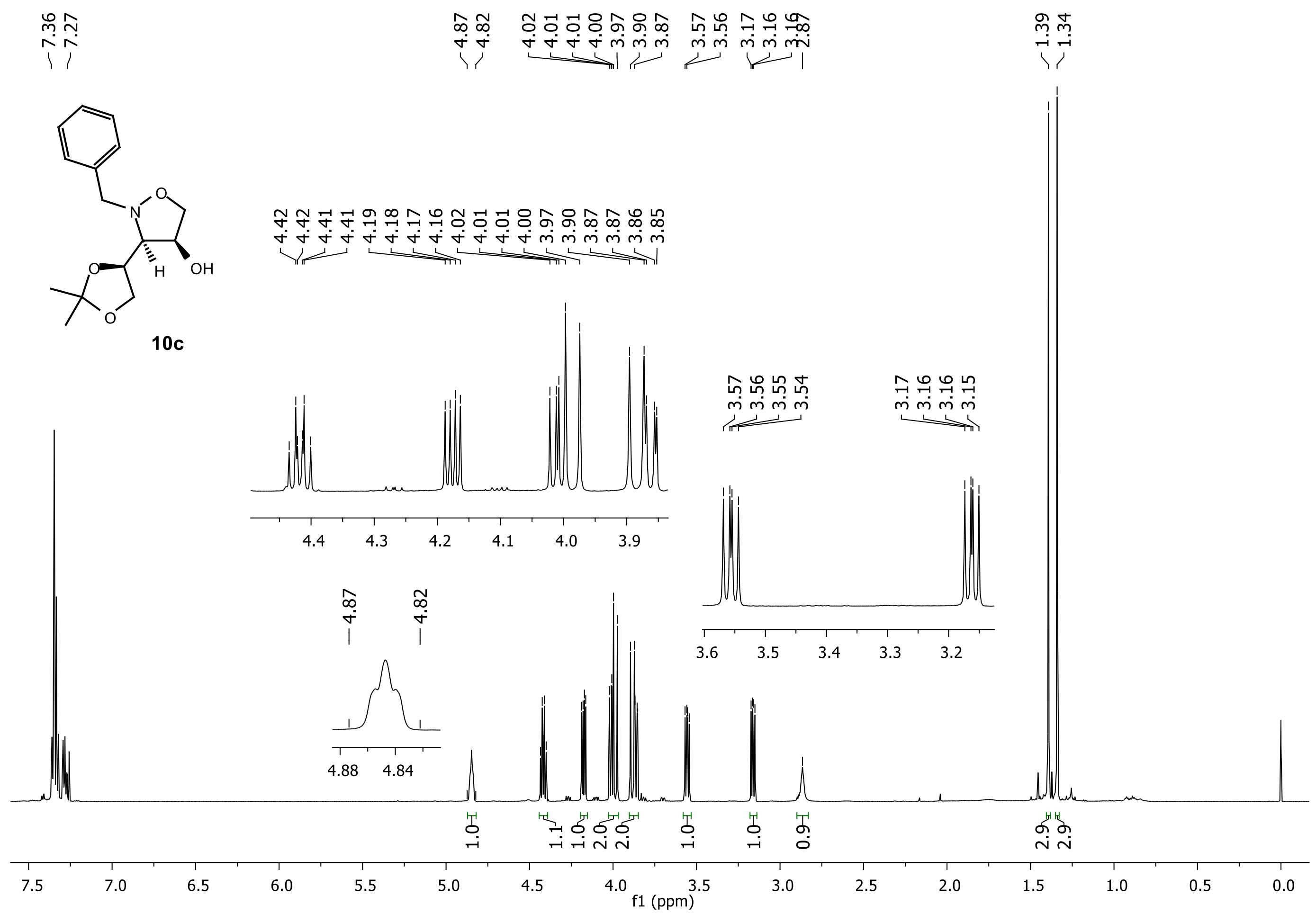
7.36
7.27
4.87
4.82
4.02
4.01
4.01
4.00
3.97
3.90
3.87
3.57
3.56
3.17
3.16
3.16
2.87
1.39
1.34
10c
OH
H
N
O
1.0
1.1
1.0
2.0
2.0
1.0
1.0
0.9
2.9
2.9
f1 (ppm)

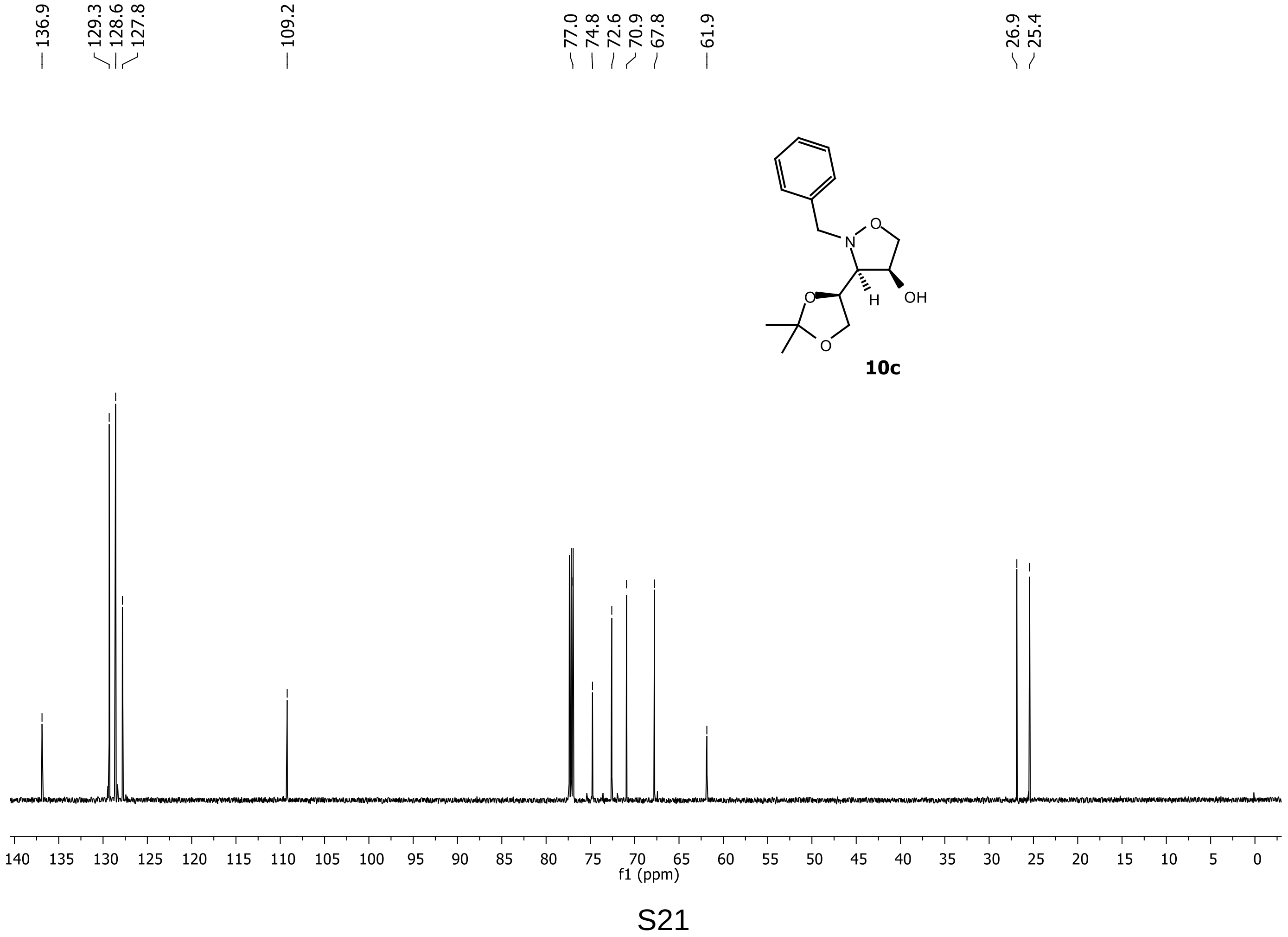
136.9
129.3
128.6
127.8
109.2
77.0
74.8
72.6
70.9
67.8
61.9
26.9
25.4
10c
OH
H
N
O
f1 (ppm)

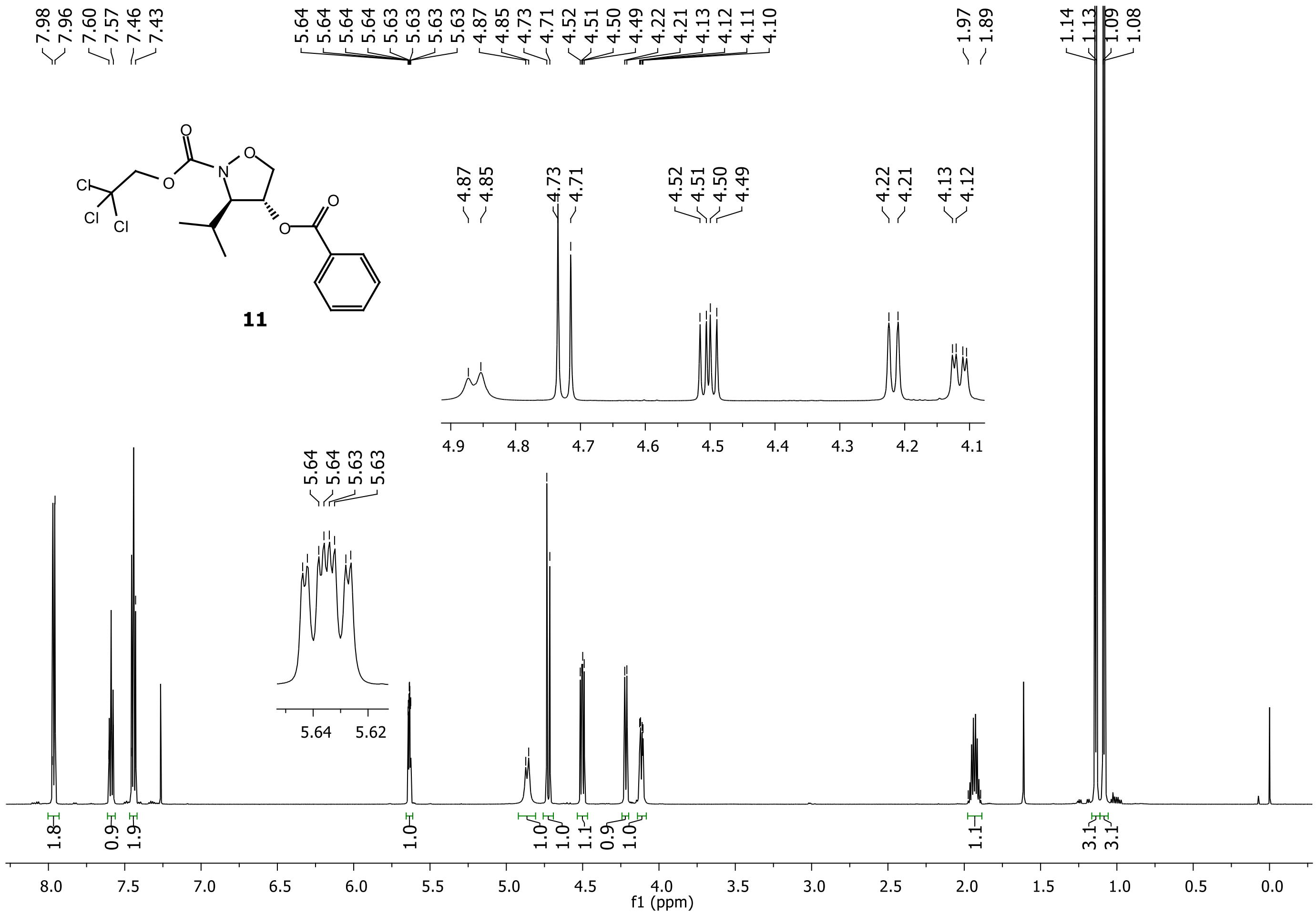
7.98
7.96
7.60
7.57
7.46
7.43
5.64
5.64
5.64
5.64
5.63
5.63
5.63
5.63
4.87
4.85
4.73
4.71
4.52
4.51
4.50
4.49
4.22
4.21
4.13
4.12
4.11
4.10
1.97
1.89
1.14
1.13
1.09
1.08
O
N
O
O
Cl
Cl
Cl
O
O
11
1.8
0.9
1.9
1.0
1.0
1.0
1.1
0.9
1.0
1.1
3.1
3.1
f1 (ppm)

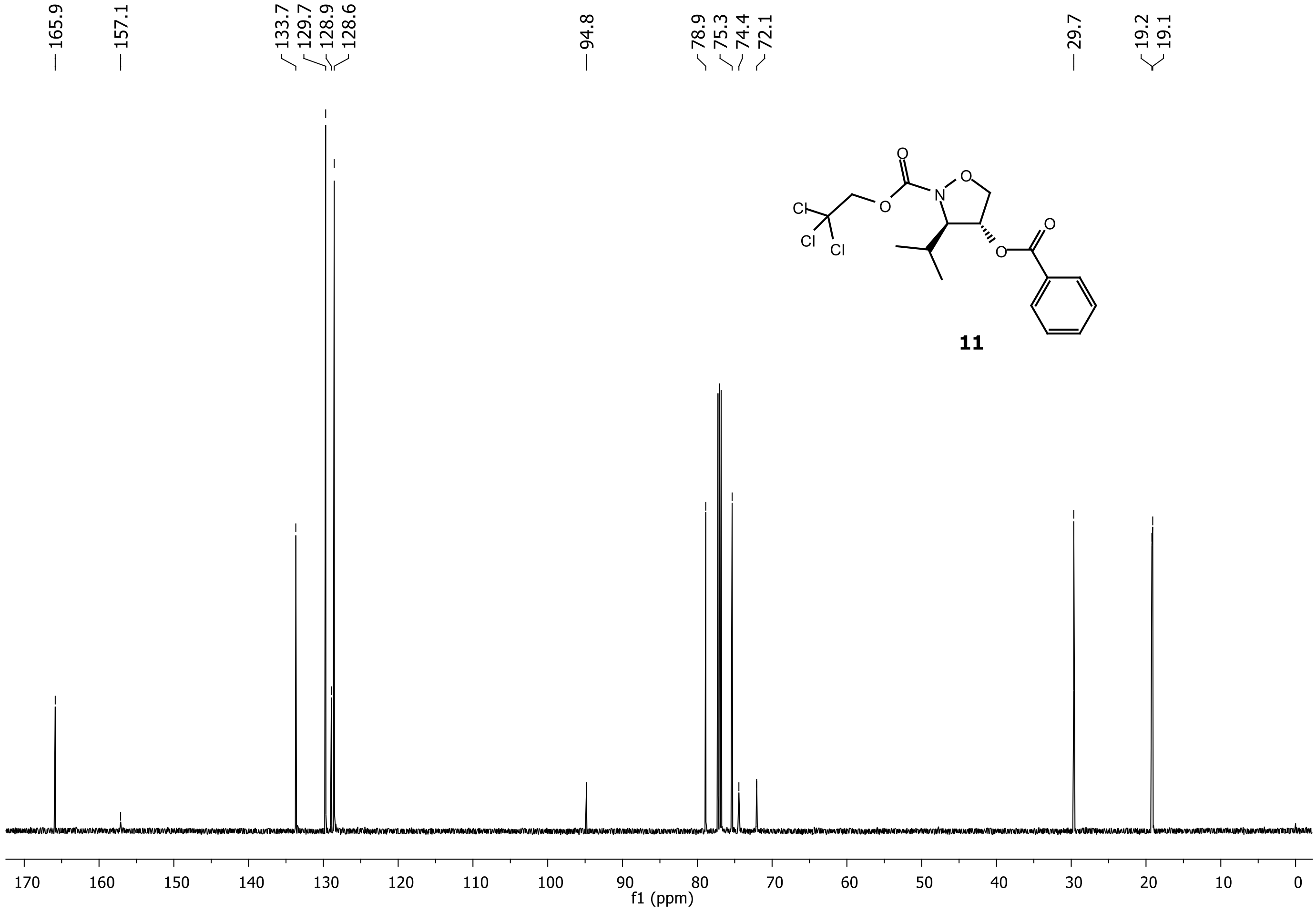
165.9
157.1
133.7
129.7
128.9
128.6
94.8
78.9
75.3
74.4
72.1
29.7
19.2
19.1
11
f1 (ppm)

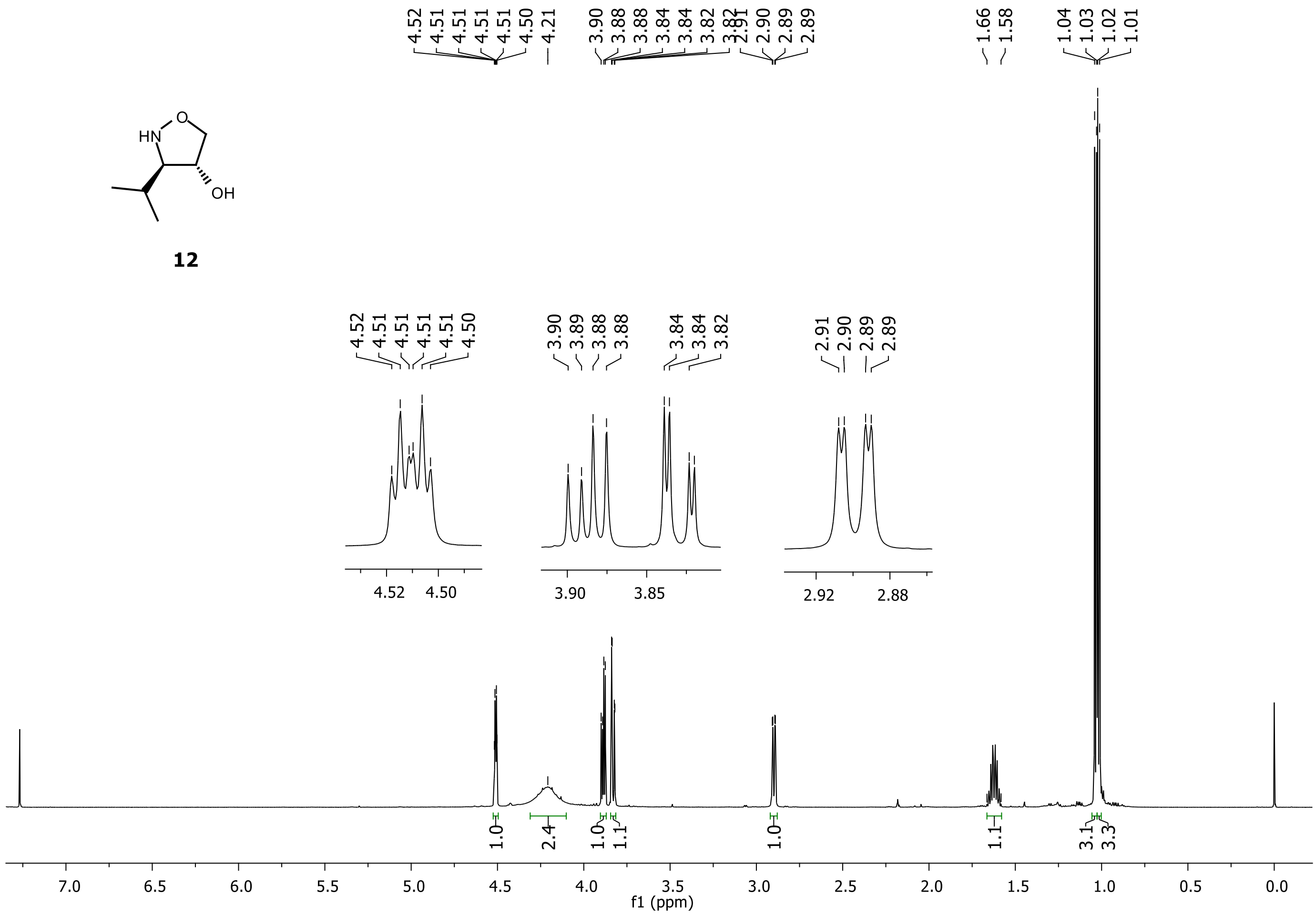
4.52
4.51
4.51
4.51
4.51
4.50
4.21
3.90
3.88
3.88
3.84
3.84
3.82
2.91
2.90
2.89
2.89
1.66
1.58
1.04
1.03
1.02
1.01
HN
O
OH
12
1.0
2.4
1.0
1.1
1.0
1.1
3.1
3.3
f1 (ppm)

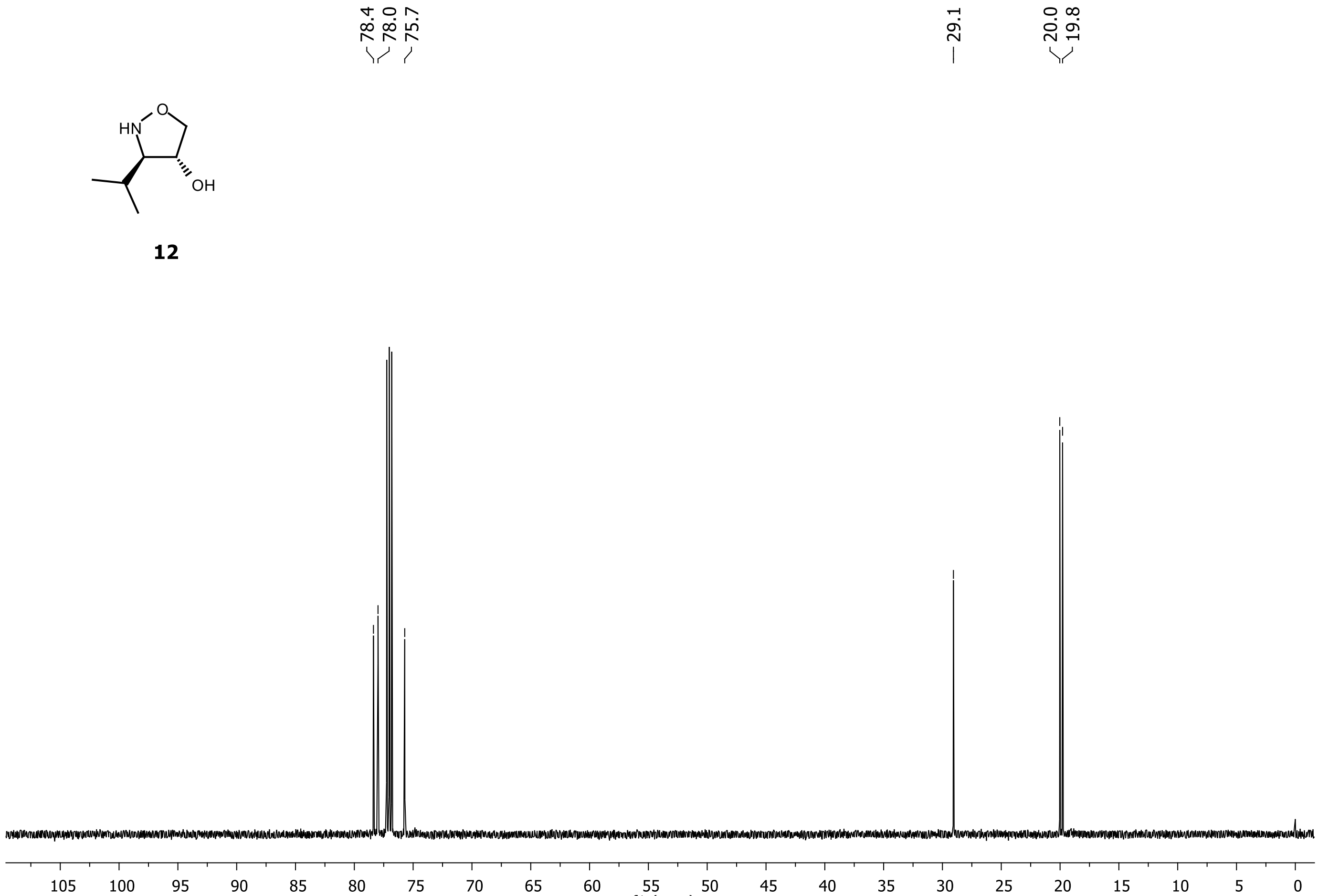
78.4
78.0
75.7
29.1
20.0
19.8
HN
O
OH
12
f1 (ppm)

References

[1] Fischer, R.; Lackovičová, D.; Fišera, L. *Synthesis* **2012**, *44*, 3783–3788.

[2] Beňadiková, D.; Čurillová, J.; Lacek, T.; Rakovský, E.; Moncoľ, J.; Doháňošová, J.; Fischer, R. *Tetrahedron* **2014**, *70*, 5585–5593.
